# Polydatin protects against DSS-induced ulcerative colitis via Nrf2/Slc7a11/Gpx4-dependent inhibition of ferroptosis signalling activation

**DOI:** 10.3389/fphar.2024.1513020

**Published:** 2025-01-14

**Authors:** Shimin Zheng, Jianbin Yin, Bingbing Wang, Qiujuan Ye, Jialuo Huang, Xinzhi Liang, Junfeng Wu, Hui Yue, Ting Zhang

**Affiliations:** ^1^ Department of Gastroenterology, The Third Affiliated Hospital of Southern Medical University, Guangzhou, China; ^2^ Department of Orthopedics, The Third Afffliated Hospital of Southern Medical University, Guangzhou, China; ^3^ Department of Gastroenterology, Nanfang Hospital, Southern Medical University, Guangzhou, China

**Keywords:** polydatin, DSS-induced ulcerative colitis, ferroptosis, Nrf2, SLC7A11, GPx4

## Abstract

**Introduction:**

Ulcerative colitis (UC), a form of inflammatory irritable bowel disease, is characterized by a recurrent and persistent nonspecific inflammatory response. Polydatin (PD), a natural stilbenoid polyphenol with potent properties, exhibits unexpected beneficial effects beyond its well-documented anti-inflammatory and antioxidant activities. In this study, we presented evidence that PD confers protection against dextran sodium sulfate (DSS)-induced ulcerative colitis.

**Methods:**

The protective effect of PD on colitis was examined in cultured caco-2 cells and DSS-induced colitis mouse model. Bulk RNA sequencing and differential gene expression analysis were used to investigate the protective mechanism of PD on DSS-induced colitis. Ferroptosis was determined by MDA levels, SOD levels, mitochondrial iron accumulation and ROS production. Ferroptosis-related proteins Slc7a11, Nrf2 and Gpx4 levels were measured by western blot, immunohistochemical and immunofluorescence staining.

**Results:**

PD mitigated the DSS-induced increases in pro-inflammatory cytokines (IL-6, TNF-α, and IL-1β), alleviated colon length shortening, reduced morphological damage to the intestinal mucosa, and preserved tight junction proteins (TJ) occludin and Zonula occludens-1 (ZO-1) in both caco-2 cells and murine models of colitis. Mechanistically, PD reversed the reduction of Nrf2, Slc7a11 and Gpx4, the degree of nuclear translocation of Nrf2 induced by DSS *in vitro* and *in vivo* significantly. Moreover, the protective effect of PD is attenuated by erastin and resembled that of Fer-1 in caco-2 cells model.

**Discussion:**

Our study suggested that PD protects against DSS-induced ulcerative colitis via Nrf2/Slc7a11/Gpx4-dependent inhibition of ferroptosis signalling activation. Further investigation into the precise mechanisms underlying this phenomenon is warranted. The findings presented herein indicated that PD may serve as a potential therapeutic agent for patients with UC.

## Introduction

Ulcerative colitis (UC) is a worldwide chronic, idiopathic, and inflammatory disorder of the rectal and colonic mucosa. In 2023, the prevalence of ulcerative colitis was estimated at 5 million cases globally, and the incidence is on the rise worldwide (Le Berre et al., 2023). A combination of immune responses, gut microbiota dysbiosis, and individual genetic predisposition contributes to the occurrence and development of UC; yet, as of now, the definitive mechanism remains unknown (Le Berre et al., 2023). Although a variety of treatments, including 5-aminosalicylic acid drugs, thiopurines, biologics, and small molecules, are recommended, the difficulties in maintaining clinical remission and achieving endoscopic normalization of UC continue to pose challenges. Therefore, it is of great significance to further investigate the pathophysiology of UC and its effective therapeutic drugs.

Ferroptosis, a distinct form of cell death characterized by iron-dependent lipid peroxidation, has been implicated across a diverse array of biological contexts, including development of aging, immunity, and cancer (Stockwell, 2022). Moreover, ferroptosis is associated with numerous organ injuries (Huang et al., 2021; Li et al., 2022) and inflammatory pathologies (Dou et al., 2022; Yang et al., 2022; Chen et al., 2023; Deng et al., 2023). Recent studies have elucidated that immunological impairment triggers alterations in inflammatory mediators and links ferroptosis to the detrimental accumulation of reactive oxygen species (ROS). The pathological setting of UC gives rise to an imbalance among lipid peroxidation, ROS levels, and antioxidant concentrations, promoting ferroptosis and contributing to its pathogenesis and advancement by triggering the death and erosion of intestinal epithelial cells (Chen et al., 2020; Long et al., 2024). Ferroptosis is controlled by a complicated network encompassing iron homeostasis, lipid metabolism, and the oxidative-reductive system. Among the diverse cellular antioxidative defense systems, the metabolic pathway of system Xc^–^-GSH-Gpx4 plays a crucial part in regulating ferroptosis (Sun et al., 2023). Solute carrier family 7 member 11(Slc7a11) is a transmembrane protein which transports extracellular cystine into the cell for the synthesis of glutathione (GSH). GSH, a crucial intracellular reductant, is capable of aiding Gpx4 in converting lipid peroxides into non-toxic lipid alcohols (Ursini and Maiorino, 2020). Hence, inadequate expression of Slc7a11 is inclined to a decreased synthesis of GSH and an accumulation of lipid peroxides, ultimately resulting in cell ferroptosis. Additionally, Nrf2, an antioxidant transcription factor, mainly governs cellular redox homeostasis, and a reduction in its transcriptional activity can induce cell ferroptosis (Dodson et al., 2019). Altering ferroptosis pathways using genetic and pharmacological approaches has been shown to affect ferroptosis. However, up to the present, nearly all the studies related to *in vivo* ferroptosis were founded on preclinical animal models, and several challenges prevent their translation into clinical practice (Sun et al., 2023). Poor pharmacokinetics continues to be a major obstacle for the further advancement of both ferroptosis antagonists like Fer-1 and Lip-1 (Sun et al., 2023). Consequently, suppressing ferroptosis in enterocytes might be a potential mechanism for treating UC, and new and more effective drugs are in urgent need of research.

Polydatin (PD), a principal constituent of *Polygonum cuspidatum* Sieb. et Zucc. (*Polygonaceae*), is extensively utilized in traditional medicine across various countries, particularly in China and Japan (Du et al., 2013; Karami et al., 2022). A growing body of evidence gathered by researchers suggests that polydatin possesses a diverse range of therapeutic effects, including antitumor (Guo et al., 2023), cardioprotective (Xie et al., 2022; Zhang et al., 2023), anti-diabetic (Kim et al., 2021), neuroprotective properties, as well as beneficial impacts on the renal system (Zhou et al., 2022), respiratory system (Yang et al., 2023) and rheumatoid diseases (Oliviero et al., 2021) across multiple disorders. PD is associated with oxidative stress and anti-inflammatory mechanisms (Zhao et al., 2018; Du et al., 2023), reducing pro-inflammatory and increasing anti-inflammatory cytokines in cases of arthritis (Du et al., 2023). Whereas a number of molecular events triggered by oxidative stress are overlapped with the process of ferroptosis, and there exist common molecular targets, like the depletion of GSH and lipid peroxidation (Chen et al., 2023). Additionally, recent investigations have revealed that PD is capable of suppressing ferroptosis to mitigate acute kidney injury (Zhou et al., 2022) as well as brain injury (Huang et al., 2021) via maintenance of the system Xc^−^-GSH-Gpx4 axis and iron metabolism. Furthermore, reports indicate that PD can be used for pain management by alleviate gut dysbiosis in irritable bowel syndrome (IBS) patients (Cremon et al., 2017; Di Nardo et al., 2024) and attenuate damage to the intestinal epithelial barrier in rats suffering from colitis (Ebrahim and Elsherbini, 2021). Nevertheless, whether the intestinal protective effect of PD is related to anti-ferroptosis remains unclear.

All the above evidence strongly suggests that PD could be a potential therapeutic compound against ferroptosis in UC. However, the antiferroptotic effect of PD on UC has not yet been clearly reported. In this study, we demonstrated that PD effectively protects against DSS-induced ulcerative colitis both *in vitro* and *in vivo*, elucidating its potential mechanism through Nrf2/Slc7a11/Gpx4-dependent inhibition of ferroptosis signaling pathways.

## Materials and methods

### Cell culture and treatments

Caco-2 cells were obtained from American Type Culture Collection and maintained in Dulbecco’s modified Eagle medium (DMEM), supplemented with 100 U/mL penicillin, 100 mg/mL streptomycin and 20% (v/v) foetal bovine serum (FBS), cultured under standard cell culture conditions of 37°C, 5% CO2, and 95% humidity. Cells were seeded in a concentration of 10^5^ cells/cm^2^. Medium were changed every 3 days. At 80% confluence, typically after 4–5 days, the cells were split 1:10 before further cultivation and passaged using 0.05% trypsin. The content of dead cells should not exceed 5% (Lea, 2015).

The cells were incubated with concentration gradient 0–90 μM PD for 48 h. Then the CCK-8 reagent was added and co-incubated for 1 h before the absorbance was measured at 450 nm on a microplate reader. In accordance with the outcomes CCK8 experiment, we employed a DSS concentration of 30 μM for the *in vitro* intervention experiment.

Prior to blood collection, immersed the tail of the mouse in warm water at approximately 50°C for several minutes to ensure the vascular engorgement of the tail. Anesthetized the animal, wiped the mouse tail with 75% alcohol, and made a transverse incision at the tail with a sharp blade to rupture the tail vein. Allowed the blood to freely drip into the container. Once the blood collection is completed, disinfected the wound and applied pressure for hemostasis. Approximately 0.1 mL of blood can be obtained from each mouse each time. A total of 0.5 mL of blood was collected from each mouse. Serum was isolated by centrifuged at 2000 rpm for 5 min.

The subsequent groups were tested: (1) Control group: the standard incubator became the place where the cells were preserved; (2) DSS group: the cells were treated with 3%DSS at 37°C for 8 h; (3) DSS + PD group: 30 μM PD pre-treatment for 2 h, followed by coprocessing with 3% DSS for 8 h; (4) DSS + PD + Erastin group: with pretreatment, the cells were treated with 5 μM erastin at 37°C for 3 h prior to DSS + PD treatment; (5) DSS + Fer-1 group: the cells were treated with 10 μM Fer-1 at 37°C for 2 h before the DSS treatment. The application of inducers (erastin) and inhibitors (Fer-1) is crucial in the study of ferroptosis and related diseases (Du and Guo, 2022).

### Animals

Wild-type male C57BL/6 mice (6–8 weeks, 20–23 g in weight) were housed in specific pathogen-free conditions. They were housed in a laboratory with controlled conditions (21°C ± 2°C temperature and 50% ± 5% humidity) and were maintained on a 12-hour light/dark cycle. The mice had access to a standard laboratory diet and sterile water *ad libitum* during the entire experimental period. The animals were acclimated to the Experimental Animal Laboratory for 10 days before the study was initiated. All animal protocols used in this study were approved and experimental procedures were conducted strictly in accordance with the Guide for the Care and Use of Laboratory Animals.

### Animal grouping and treatments

The mice were randomly divided into three groups (*n* = 5 mice per group): a control group, a DSS group and a DSS plus 30 mg/kg/day PD group (Jiang et al., 2017; Lv et al., 2018). DSS-induced colitis model was prepared according to the methods reported by Stefan et al. (2017) and Yao et al. (2011). The PD concentration of action was determined based on previous studies (Jiang et al., 2017; Lv et al., 2018). Ulcerative colitis was induced by administrating 3% DSS for 7 days. PD was administered via gavage for 7 days in parallel with DSS. Mice were given drinking water for 2 days after DSS and/or PD treatment. Weight loss, stool characteristics, and fecal occult blood were recorded during the progression of the DSS-induced mouse IBD model. The disease activity index (DAI) (Deng et al., 2018) was calculated as previously described. After 9 days, the animals were sacrificed by cervical dislocation.

Colon biopsy specimens were acquired and colon length was measured. Opened colon lengthwise, and the feces were removed. The specimens were washed with a 0.85% NaCl saline solution and promptly examined. Animal handling was approved by the Animal Experimental Ethics Committee of Southern Medical University (number: SMUL2022289; approval date: 20,221,214).

### Histological analysis

The distal colon was fixed in 4% paraformaldehyde, and then, they were dehydrated with a graded ethanol series: 30%, 50%, 75%, 85%, 95%, and 100% ethanol (twice) respectively; cleared in 100% dimethylbenzene twice, each for 30 min; infiltrated paraffin (52°C–54°C soft paraffin, 54°C–56°C soft paraffin, 56°C–58°C hard paraffin, for 1 h respectively); embedded in 60°C paraffin and stained with haematoxylin and eosin (H&E). The degree of intestinal inflammation was assessed according to the modified scoring system devised by Dieleman et al. (1998). The scores were assessed by two pathologists in double-blinded manner.

### Real-time quantitative polymerase chain reaction (RT-qPCR)

Total RNA was extracted from cells using Trizol (Invitrogen, CA, USA). RNA samples were subjected to RT using a First Strand cDNA Synthesis Kit (Takara, Dalian, China). We purified the RNA using our lithium chloride (LiCl) protocol to remove all polysaccharides (including DSS) from the samples (Viennois et al., 2013). The RNA was precipitated twice by 0.1 volume of 8 M LiCl, followed by a precipitation step in 0.1 volume of 3 M sodium acetate (pH 5.2) and 2 volumes of 100% ethanol. The RNA were then centrifuged, pellets were washed with 100 μL of 70% ethanol and RNAs were finally dissolved in 20–50 μL of RNase-free water. The primers used for quantitative PCR are listed in Supplementary Table S1. PCR was initiated by a 5-min incubation at 94°C, and ended after a 10-min extension at 72°C, with 40 cycles for denaturation at 94°C for 30 s, annealing at 60°C for 30 s, and extension at 72°C for 1 min using a PCR kit (SBS Gene Tech Co., Beijing, China). The results were analyzed with 480II Real-Time PCR System software (Roche). GAPDH mRNA was amplified simultaneously as an internal control. Each assay was performed in triplicate.

### Enzyme-linked immunosorbent assay (ELISA) analysis

Before collecting blood from mice, warm the tail, anesthetize, cut the tail vein for blood to drip, disinfect and stop bleeding after collection, and isolate serum by centrifugation. Serum samples were obtained from the different groups, and protein levels of the inflammatory factors TNF-α, IL-1β, IL-6 were determined using commercially available ELISA kits (MultiSciences, Hangzhou, China), according to the manufacturer’s protocol. Absorbance was read at 450 nm, and the concentration was determined by comparing their optical densities to a standard curve.

### Western blot analysis

Total protein was extracted with cold radioimmunoprecipitation lysis buffer, protease inhibitor and phosphatase inhibitor cocktail. Equal amounts of proteins were separated by 8% or 10% SDS-polyacrylamide gels and then transferred to a PVDF membrane (Bio-Rad, Marnes-laCoquette, France), which was blocked with 5% skim milk prepared in tris-buffered saline with Tween (TBST). Then, the membrane was incubated with primary antibodies against GAPDH (1:5000, ZSGB-BIO, Beijing, China), ZO-1 (1:1000, Invitrogen, Carlsbad, CA, USA), occludin (1:1000, Protein Tech Group, Inc., Wuhan, China), Nrf2 (1:1000, 16396-1-AP, Proteintech, USA), Slc7a11 (1:1000, ab307601, Abcam, United Kingdom), Gpx4 (1:1000, 67763-1-IG, Proteintech, USA) overnight at 4°C. Following washes in TBST, the membrane was subsequently incubated with the appropriate HRP-conjugated secondary antibodies (1:3000) for 1 h and then visualized via enhanced chemiluminescence detection. Protein expression was quantified by densitometric analysis using ImageJ software (National Institutes of Health, Bethesda, MD, USA). Each assay was performed in triplicate.

### The SOD activity and MDA level analysis

The SOD activity of cells at different groups was measured by a Total Superoxide Dismutase Assay Kit with WST-8 (S0101S, Beyotime, Jiangsu, China). The WST-8/enzyme working solution was reacted with the supernatants, and the reaction products were spectrophotometrically evaluated in a gauge at 450 nm and the products after the reaction were measured spectrophotometrically at 532 nm (BioTek, USA). The entire experiment was repeated in triplicate, where U/mg protein was used to represent the activity of SOD. MDA levels were measured using the Lipid Peroxidation MDA Assay Kit (S0131S, Beyotime, Jiangsu, China), which is based on the reaction of MDA and thiobarbituric acid (TBA). The products after the reaction were measured spectrophotometrically at 532 nm (BioTek, USA). The entire experiment was repeated in triplicate, where nmol/mg protein was used to represent the level of MDA.

### Reactive oxygen species (ROS) determination

For detection of reactive oxygen species, Lipid Peroxidation Assay Kit (BODIPY 581/591C11, S0043S) was utilized as instructed by the manufacturer’s protocol. Caco2 cells were incubated with 1 mL BODIPY 581/591 C11 staining working solution at 37°C for 30 min in darkness after treatment. The cells were washed twice with PBS and then collected for fluorescence microscopy (Olympus BX51) observation. Each assay was performed in triplicate.

### Detection of mitochondrial labile iron

With the help of the manufacturer’s protocol, aiming at detecting mitochondrial labile iron, the fluorophore Mito-FerroGreen was utilized accordingly. Caco2 cells were plated on 14 mm round cell slides in 24-well plates and incubated with 5 μmol/L FerroGreen (diluted in DMEM without serum) at 37°C for 30 min in lightless surroundings after treatment. By using serum-free DMEM, the cells were washed twice. Fluorescence was visualized with the support of a fluorescence microscope (Nikon A1R, Shanghai, China). Each assay was performed in triplicate.

### Immunohistochemical staining (immunoenzymological staining)

The immunohistochemical methods have been described previously. Sections were deparaffinized in xylene and rehydrated through a graded ethanol series. The detailed procedure is as follows: xylene (10 min), xylene (10 min), absolute ethanol (5 min, twice), 95% ethanol (5 min, twice), 90% ethanol (5 min), 85% ethanol (5 min), 80% ethanol (5 min), 75% ethanol (5 min). Then, they were antigen retrieved (thermal-induced epitope retrieval, 0.01M citrate sodium buffer solution 95°C, 15 min), quenched of endogenous peroxidase (3%H_2_O_2_) and incubated with primary antibodies against Occludin and ZO-1 (dilution 1:100) overnight at 4°C. After washing with PBS three times, tissues were incubated with a second antibody (ZSGBBIO), developed with the DAB reagent and counterstained with haematoxylin. Negative controls were incubated without antibody. The quantification of TJ proteins was assessed according to the staining intensity and the percentage of the stained epithelial cells. Briefly, the staining intensity was scored as negative (0), weak (1), moderate (2) or strong staining (3). The percentage of positive cells was graded on a scale of 0–4: 0, less than 5%; 5%–25% scored 1; 26%–50% scored 2; 51%–75% scored 3 and more than 75% scored 4. The final score was calculated by multiplying intensity score and percentage score.

### Immunohistochemical staining (immunofluorescence)

The specimens were dried in a 65°C oven for 2 h for dewaxing and dehydration, and then soaked in deionized water for 5 min. The tissue specimens were soaked in sodium citrate solution overnight in a 60°C water bath to expose the antigen. The sections were immersed in phosphate buffer solution (PBS) and then incubated with 10% goat serum at 37°C for 1 h for antigen blocking. The slices were incubated at 4°C for 12 h with the corresponding primary antibody, rewarmed at room temperature, and washed with PBS. The cells were incubated with 100 μL/well working solution containing Alexa Fluor 594conjugated goat anti-rabbit secondary antibody at room temperature for 1 h in the dark. 4,6-diamino-2phenylindole (DAPI; Thermo Fisher Science, United States) was used for nuclear counterstaining. The stained slides were imaged using an inverted fluorescence microscope (magnification, ×200; Olympus Corporation).

### Immunofluorescence staining

Caco-2 monolayers were fixed in 4% paraformaldehyde for 10 min at room temperature. Following permeabilization in 0.1% Triton X-100, they were blocked in PBS containing 5% bovine serum albumin for 1 h at room temperature. Then, the monolayers were incubated with a primary antibody against ZO-1 (1:50), occludin (1:50), Nrf2 (1:50) and Gpx4 (1:50) overnight at 4°C. The cells were subsequently incubated with the appropriate secondary fluorescence antibody for 1 h. 5 μg/mL DAPI was used to stain the cell nuclei. The fluorescence was examined under a confocal laser scanning microscope (Fluoview FV10i; Olympus, Tokyo, Japan). Each assay was performed in triplicate.

### Bulk RNA sequencing and diff-gene analysis

Total RNA was extracted from the samples by Trizol reagent (Invitrogen) separately. The RNA quality was checked by Agilent 2,200 and kept at −80°C. The RNA with RIN (RNA integrity number) > 7.0 is acceptable for cDNA library construction. The cDNA libraries were constructed for each RNA sample using the VAHTS Universal V6 RNA-seq Library Prep Kit for Illumina (vazyme, Inc.) according to the manufacturer’s instructions. Generally, the protocol consists of the following steps: Poly-A containing mRNA was purified from 1ug total RNA using oligo (dT) magnetic beads and fragmented into 200–600 bp using divalent cations at 85°C for 6 min. The cleaved RNA fragments were used for first- and second-strand complementary DNA (cDNA) synthesis. dUTP mix was used for second-strand cDNA synthesis, which allows for the removal of the second strand. The cDNA fragments were end repaired, A-tailed and ligated with indexed adapters. The ligated cDNA products were purified and treated with uracil DNA glycosylase to remove the second-strand cDNA. Purified first-strand cDNA was enriched by PCR to create the cDNA libraries. The libraries were quality controlled with Agilent 2,200 and sequenced by DNBSEQ-T7 on a 150 bp paired-end run.

Before read mapping, clean reads were obtained from the raw reads by removing the adaptor sequences and low-quality reads (Fastp, version: 0.19.7, https://github.com/OpenGene/fastp). The clean reads were then aligned to Human genome (GRCh38, Ensembl) using the Hisat2 (Kim et al., 2015). HTseq (Anders et al., 2015) was used to get gene counts and RPKM method was used to determine the gene expression.

We applied EBSeq algorithm (Leng et al., 2013) to filter the differentially expressed genes, after the significant analysis, *P*-value and FDR analysis (Benjamini et al., 2001) were subjected to the following criteria: i) Fold Change>1.5 or <0.667; ii) *P*-value<0.05, FDR<0.05.

Gene ontology (GO) analysis was performed to facilitate elucidating the biological implications of the differentially expressed genes in the experiment (Ashburner et al., 2000). We downloaded the GO annotations from NCBI (http://www.ncbi.nlm.nih.gov/), UniProt (http://www.uniprot.org/) and the Gene Ontology (http://www.geneontology.org/). Fisher’s exact test was applied to identify the significant GO categories (*P*-value <0.05).

Pathway analysis was used to find out the significant pathway of the differentially expressed genes according to KEGG database. We turn to the Fisher’s exact test to select the significant pathway, and the threshold of significance was defined by *P*-value< 0.05.

### Statistical analysis

Analysis of all data was obtained based on the statistical software GraphPad Prism (version 5.0). All data were representative of at least three independent experiments and were expressed as the mean ± S.E.M. Groups were compared by one-way analysis of variance followed by the least significant difference test (*p < 0.05, **p < 0.01, ***p < 0.001). Multiple comparison between the groups was performed using Tukey’s test for *post hoc* analysis. Three replicates were conducted for each group in this study.

## Results

### Polydatin alleviates DSS-induced colitis in Caco-2 cells

**FIGURE 1:**
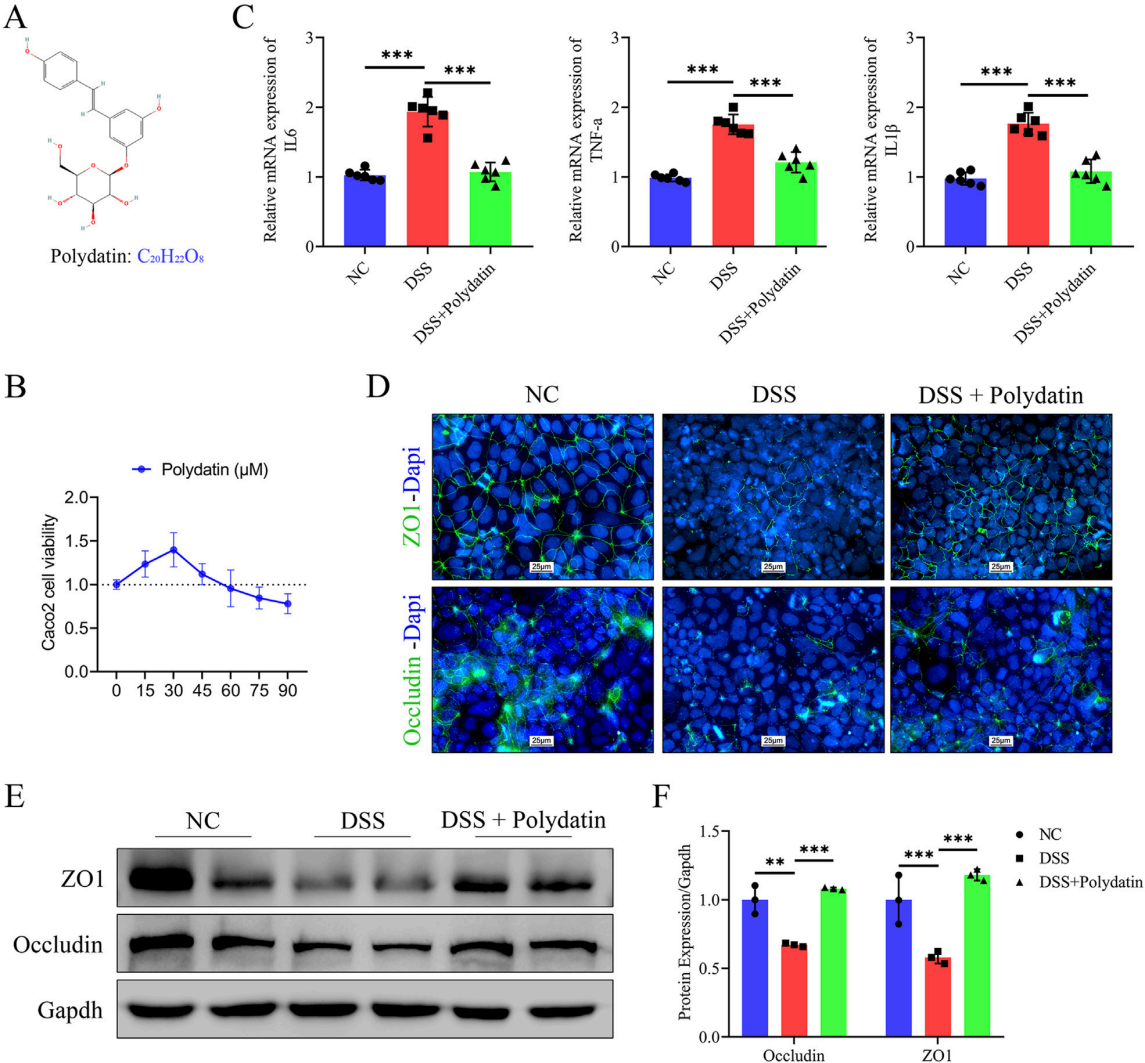
The protective effects of polydatin (PD) on DSS-induced ulcerative colitis *in vitro*. **(A)** Chemical structure of PD. **(B)** Caco-2 cells viability was evaluated by CCK-8 assay in medium after 0–90 μM PD treatment for 48 h. **(C)** The mRNA levels of IL-6, TNFα and IL-1β in caco-2 cells. **(D–F)** Expression levels of TJ proteins ZO-1 and occludin were determined by immunofluorescence and Western blotting. Band densities were evaluated by ImageJ software. The cell nuclei were stained with DAPI (blue); ZO-1 and occluding were stained green; NC, normal control; **P < 0.01; ***P < 0.001.

The chemical structure of polydatin is depicted in Figure 1A. As revealed by the CCK-8 assay, PD (0–30 μM) do not exert an inhibitory effect on the activity of caco-2 cells. Therefore, we select the maximum concentration 30 μM as the experimental concentration (Figure 1B). To verify whether PD can confer protection against DSS-induced UC, we initially treated Caco-2 cell monolayers with either 3% DSS or a combination of 3% DSS and 30 μM PD and subsequently assessed the mRNA levels of pro-inflammatory cytokines IL-6, TNF-α, and IL-1β in Caco-2 cells (Figure 1C). Treatment with PD at 30 μM significantly inhibited the expression of these cytokines (DSS + Polydatin vs. DSS, *P* < 0.001). As intestinal epithelial barrier function and inflammation are intimately related, we also investigated the levels of TJ proteins ZO-1 and occludin. Immunofluorescence staining demonstrated that DSS treatment led to depletion and discontinuity of both ZO-1 and occludin. PD was capable of attenuating these changes (Figure 1D). The levels of TJ proteins ZO-1 and occludin were significantly decreased upon DSS exposure (DSS vs normal control; occludin, *P* < 0.01; ZO-1, *P* < 0.001), and they were reversed when combined with PD treatment (Figures 1E, F, DSS + Polydatin vs DSS; *P* < 0.001).

### Polydatin ameliorates the severity of DSS-induced colitis in mice

**FIGURE 2:**
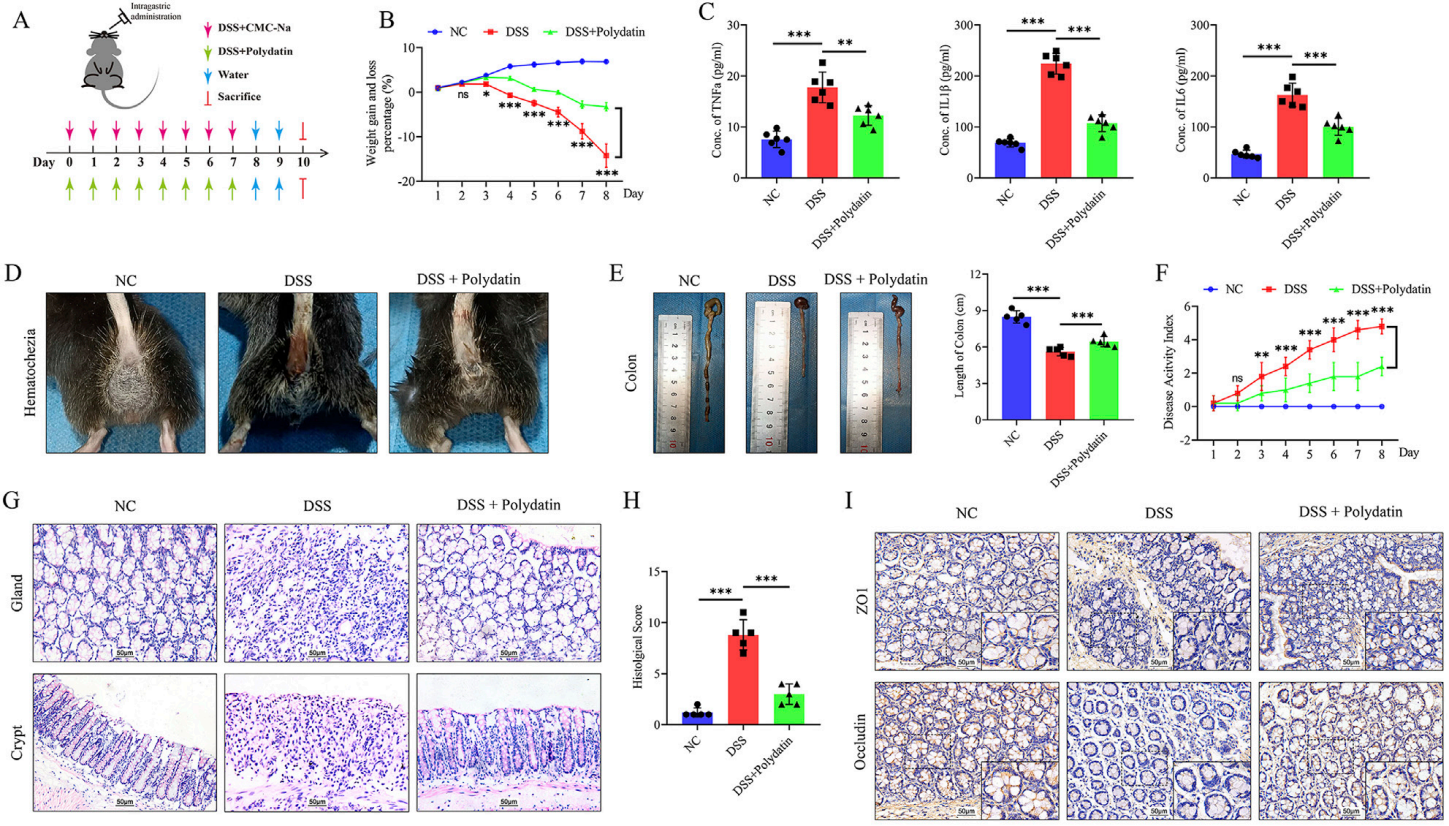
PD alleviates the severity of DSS-induced ulcerative colitis in mice. C57BL/6 mice were given 3% DSS to induce acute colitis. The Polydatin group was administered 30 mg/kg/d Polydatin dose. **(A)** Protocol for the treatment of mice. **(B)** Bodyweight. **(C)** The levels of IL-6, TNF-α and IL-1β in serum, as demonstrated by ELISA. **(D)** hematochezia. **(E)** Colon length. **(F)** DAI scores. **(G)** Histological analysis, H&E staining of the proximal colon; 200×. **(H)** Quantitation of histological scores. **(I)** Immunohistochemical staining of occludin and ZO-1 in colon of mice, overview: ×200; magnification: ×400. NC, normal control; ns, no significance; *P < 0.05, **P < 0.01, ***P < 0.001, DSS+Polydatin versus DSS.

To ascertain whether PD exerts beneficial effects in colitis, we administered 3% DSS to induce acute colitis in mice (Figure 2A). Mice concurrently treated with PD manifested significantly less body weight loss than those treated solely with DSS (Figure 2B; DSS + Polydatin vs. DSS; ns, no significance; **P < 0.01; ***P < 0.001. We evaluated levels of pro-inflammatory cytokines IL-6, TNF-α, and IL-1β in serum and discovered that PD could significantly suppress the expression of these cytokines (Figure 2C; DSS + Polydatin vs DSS; *P* < 0.001). Consistent with these findings, PD conspicuously reduced colon length shortening (Figure 2E) as well as the disease activity indices (DAIs) of DSS-induced mice (Figures 2D, F, DSS + Polydatin vs DSS; ns, no significance; **P < 0.01; ***P < 0.001). Moreover, histological examination disclosed less intestinal mucosal morphological damage, a reduced infiltration of inflammatory cells, and superior histological scores in PD-treated mice (Figures 2G, H, DSS vs normal control; *P* < 0.001; DSS + Polydatin vs DSS; *P* < 0.001). Additionally, we found that the expression of TJ proteins in mice undergoing PD treatment was higher than that in mice treated only with DSS (Figure 2I), suggesting that PD could attenuate intestinal barrier dysfunction and ulcerative colitis.

### In polydatin protect against DSS-induced colitis model, ferroptosis and Slc7a11 are of extreme significance

**FIGURE 3:**
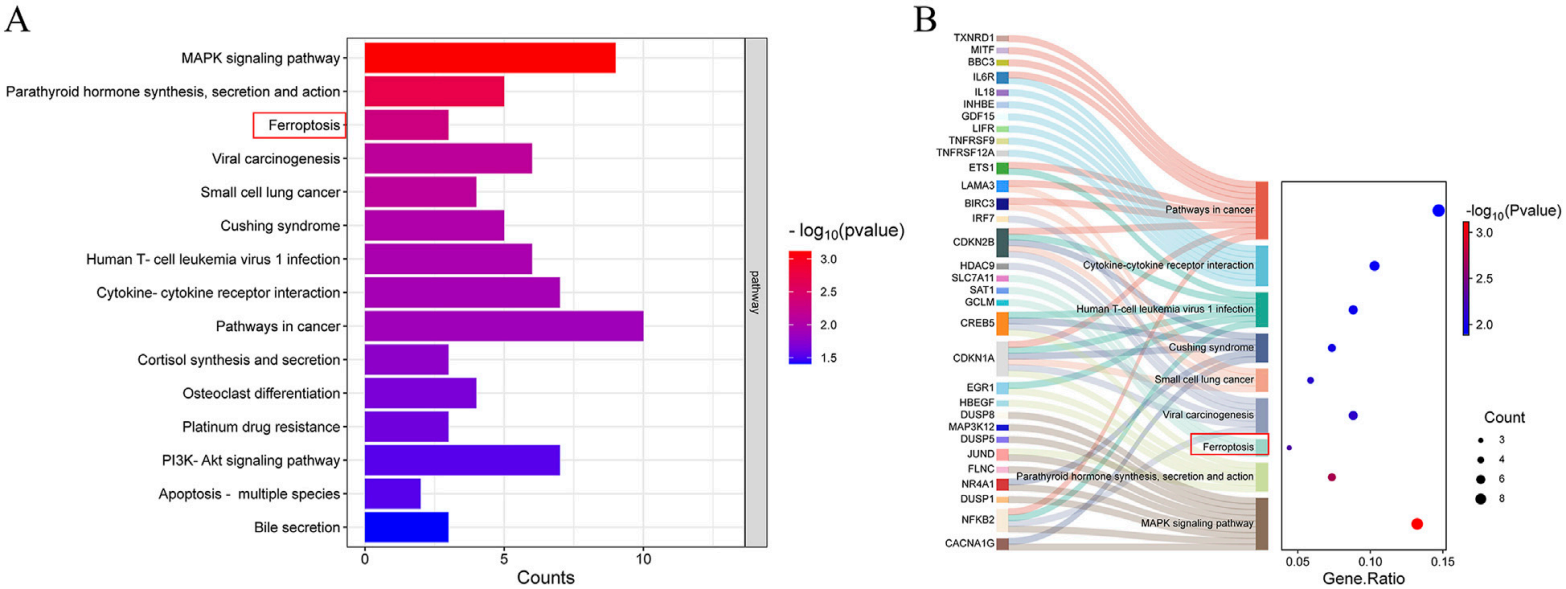
Ferroptosis and Slc7a11 are vital in the protective effects of PD on UC. Bulk RNA sequence was performed on caco-2 cells treated with 3% DSS and 3%DSS+30 μM PD. **(A)** Pathway enrichment analysis of different genes. **(B)** The Sankey bubble diagram. Y-axis: pathways through which various molecules exert their functions; X-axis: gene ratio, the proportion of the number of genes in the target pathway enriched from the gene list to the total number of genes included in the gene set. Bubble size: number of enriched genes; Bubble color: reflects the significance of enrichment (the size of the *P*-value).

RNA sequencing and subsequent data analysis have enabled researchers to further illuminate the functional complexity of transcription. Hence, we utilized bulk RNA sequencing to computationally investigate the protective mechanism of PD in ulcerative colitis. Total RNA was extracted from Caco-2 cells treated with either 3% DSS or a combination of 3% DSS and 30 μM PD for RNA sequencing and differential gene analysis. The results of pathway enrichment analysis indicated that the expressions of ferroptosis-related regulatory factors in the DSS + PD group were significantly augmented, suggesting the probable involvement of ferroptosis in the pathological process during PD’s mitigating effect on UC (Figure 3A). The Sankey bubble diagram demonstrated a significant upregulation in the expression of Slc7a11, SAT1, and GCLM within the ferroptosis pathway (Figure 3B). Among these regulatory factors, Slc7a11 has been reported as one of the most crucial targets, whose inhibition is known to induce ferroptosis (Seibt et al., 2019).

### Polydatin exerts a protective effect on caco-2 cells by inhibiting ferroptosis via Nrf2/Slc7a11/Gpx4 signaling pathway

**FIGURE 4:**
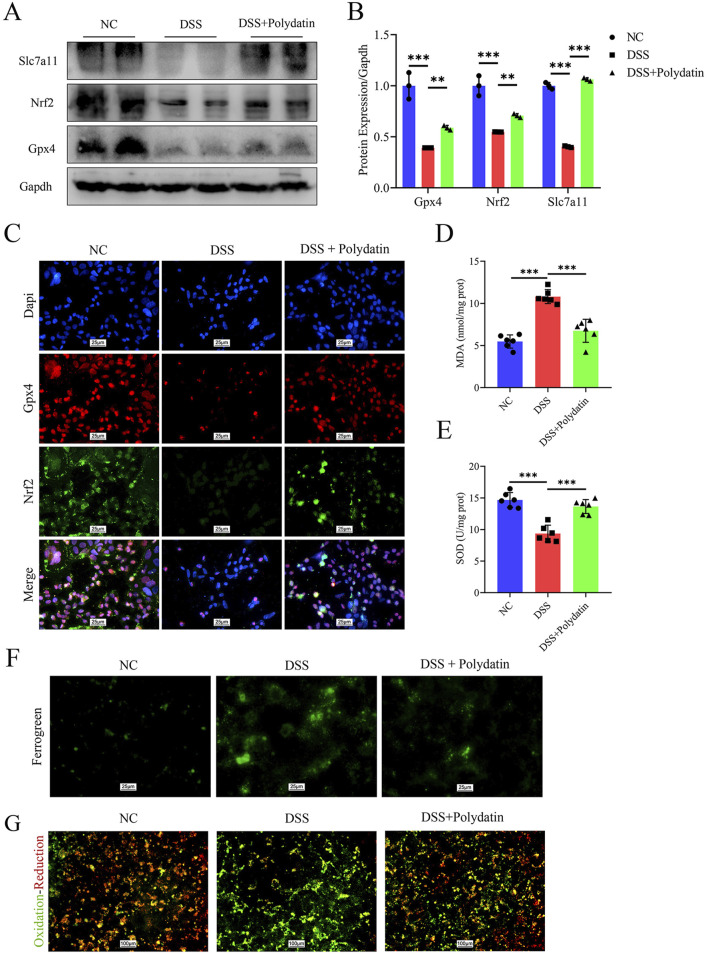
PD exerts a protective effect on caco-2 cells by inhibiting ferroptosis through the Nrf2/Slc7a11/Gpx4 signaling pathway. After pre-treatment with 30 μM Polydatin for 2 h, the Caco-2 cell monolayers were co-stimulated with 3% DSS for 8 h **(A, B)** Western blotting detected the levels of Slc7a11, Nrf2 and Gpx4 in caco-2 cells, and band densitometric analysis was performed. **(C)** Co-expression of Nrf2 and Gpx4, along with the immunofluorescence staining analysis of Nrf2 nuclear translocation. Dapi, blue; Gpx4, red; Nrf2, green. **(D, E)** MDA and SOD levels in caco-2 cells. **(F, G)** Mitochondrial ferrous ions aggregation and ROS production in caco-2 cells. **P < 0.01; ***P < 0.001.

The activation of ferroptosis is involved in DSS-induced colitis (Chen et al., 2020). The prevention of colitis by PD might be related to ferroptosis and the upregulation of Slc7a11 expression. Slc7a11 and Gpx4, two of the most crucial targets whose inhibition induces ferroptosis, are well regulated by Nrf2 (Sato et al., 1999; Yang et al., 2014). Therefore, in the next step, we explored the alterations in components of ferroptosis *in vitro* by means of Western blot assay. As shown in Figures 4A, B, the reduction of Nrf2, Slc7a11, and Gpx4 induced by DSS in Caco-2 cells was significantly reversed by PD treatment (DSS vs. normal control, ***P < 0.001; DSS + Polydatin vs DSS, **P < 0.01; ***P <0.001). We also investigated the co-expression of Nrf2 and Gpx4, as well as the nuclear translocation of Nrf2 through immunofluorescence staining (Figure 4C). After DSS treatment, the co-expression of Nrf2 and Gpx4, as well as the degree of nuclear translocation of Nrf2, decreased significantly. However, after combined PD and DSS treatment, the expression levels increased considerably, approaching those of the control group. The levels of reactive oxygen species (ROS) were detected with the aid of cell fluorescence detection, and the results indicated that DSS treatment significantly upregulated the levels of ROS, while PD and DSS treatment partially reversed this effect (Figure 4G). Moreover, similar to the change in ROS levels, only DSS treatment significantly increased the level of mitochondrial iron (Figure 4F), along with the release of malondialdehyde (MDA) and superoxide dismutase (SOD) (Figures 4D, E, DSS vs normal control, ***P < 0.001; DSS + Polydatin vs DSS, **P < 0.01; ***P < 0.001), which was partially rescued in the DSS and PD treatment group.

### Polydatin improves DSS-induced colitis in mice by inhibiting ferroptosis via Nrf2/Slc7a11/Gpx4 signaling pathway

**FIGURE 5:**
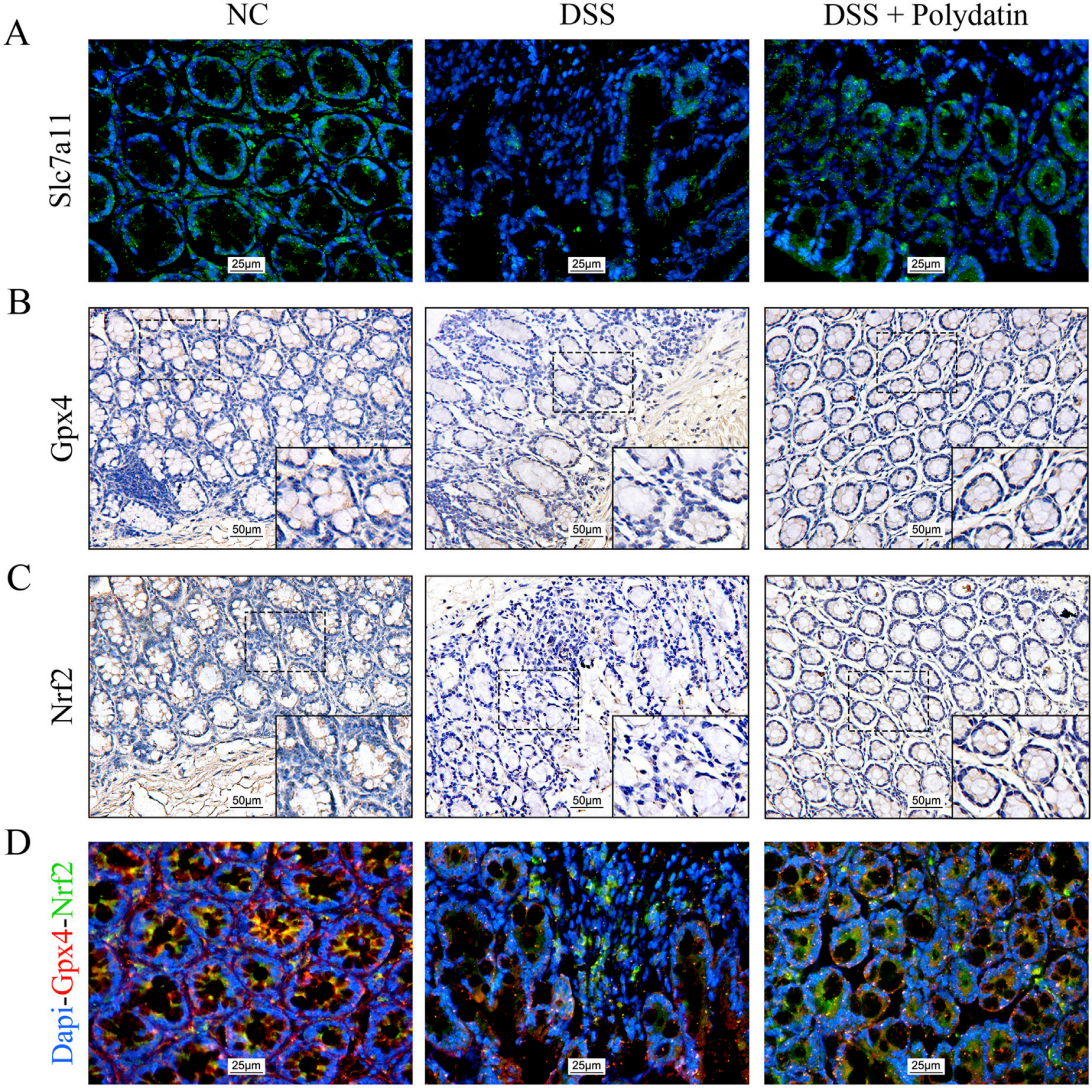
Polydatin ameliorates DSS-induced colitis in mice by inhibiting ferroptosis through the Nrf2/Slc7a11/Gpx4 signaling pathway. **(A)** Immunofluorescence staining of Slc7a11 in the colon. magnification: ×400. **(B, C)** Immunohistochemical staining of Gpx4 and Nrf2 in the colon. overview: ×200; magnification: ×400. **(D)** Immunofluorescence images of Nrf2 and Gpx4 co-expression and the Nrf2 nuclear translocation in the colon. magnification: ×400; Red: Gpx4; Green: Nrf2.

We also examined whether the colitis-protective mechanism of PD is mediated by ferroptosis through the Nrf2/Slc7a11/Gpx4 axis *in vivo*. Immunofluorescence and double immunofluorescence staining were carried out on the colorectal tissue sections of mice. The data indicated that the decreased expression of Slc7a11 and Gpx4 induced by DSS was significantly upregulated in the mice of the PD treatment group (Figures 5A, B). The nuclear translocation of Nrf2 was more severely disrupted in mice after DSS exposure, but was markedly enhanced in those treated with both DSS and PD (Figures 5C, D). Taken together, these data verified that PD ameliorates DSS-induced colitis by inhibiting ferroptosis activation via an Nrf2/Slc7a11/Gpx4-dependent signaling pathway.

### The protective effect of polydatin is attenuated by erastin and is similar to that of Fer-1 *in vitro*

**FIGURE 6:**
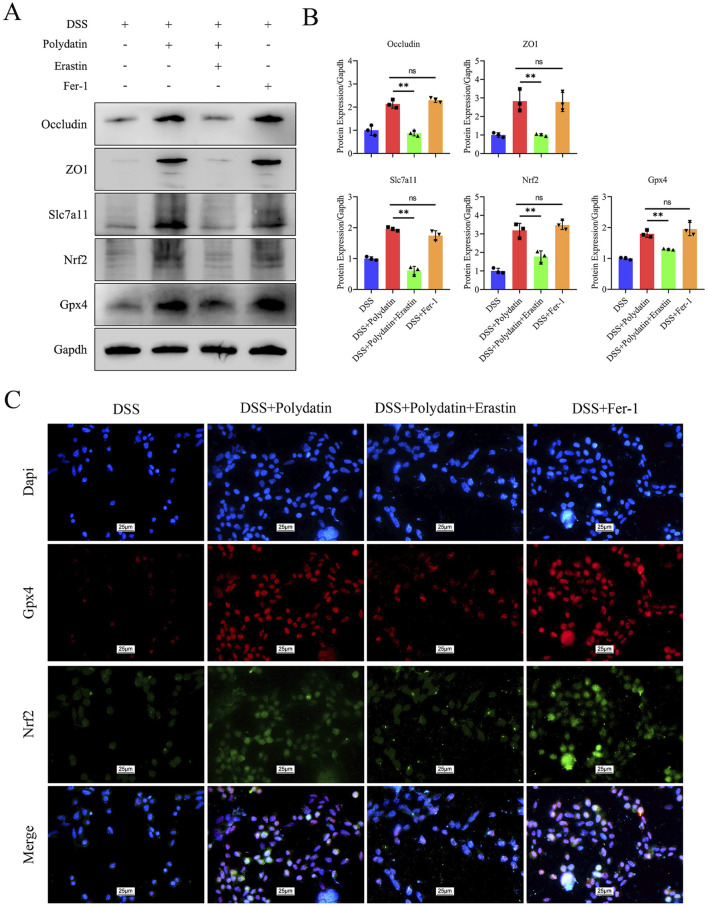
The protective efficacy of polydatin was attenuated by erastin and exhibited resemblance to that of Fer-1. **(A, B)** Levels of occludin, ZO1, Nrf2, Gpx4 and Slc7a11 in the colon assessed by Western blotting and band densitometric analysis was performed. **(C)** Co-expression of Nrf2 and Gpx4, along with the nuclear translocation of Nrf2. **P < 0.01, DSS + Polydatin vs. DSS + Polydatin + Erastin; ns, no significance, DSS + Polydatin vs. DSS + Fer-1. Dapi, blue; Gpx4, red; Nrf2, green.

To illuminate the crucial role of ferroptosis in ulcerative colitis (UC), we introduced Fer-1 and erastin treatments in combination with PD or/and DSS treatments. The results following the application of a ferroptosis inducer revealed that the expression of ZO-1, Occludin, Nrf2, Slc7a11, and Gpx4 was significantly downregulated in the erastin group compared to the PD and DSS treatment group. Intriguingly, these downregulated proteins were upregulated in both the PD group and the Fer-1 group to a comparable extent. There was no significant difference between the PD group and the Fer-1 group, suggesting that the inhibitory effect of PD on ferroptosis was analogous to that of Fer-1 and that PD exerted its protective effect by inhibiting ferroptosis (Figures 6A, B, DSS + Polydatin + Erastin vs. DSS + Polydatin, ***P < 0.001; DSS+Fer-1 vs DSS+Polydatin, no significance). Similar to the changes observed in the Western blot analysis, the co-expression of Nrf2 and Gpx4 as well as the nuclear translocation of Nrf2 were significantly upregulated in the Fer-1 + DSS group and the PD + DSS group, but downregulated in the erastin + PD + DSS group (Figure 6C).

## Discussion

In this study, we presented evidence that PD protected against DSS-induced colitis by modulating the expression of ferroptosis-related proteins and their nuclear translocation both *in vitro* and *in vivo*. This suggests innovative strategies for utilizing PD as a potential treatment for UC. The underlying mechanism behind this protective effect may involve activation of the Nrf2/Slc7a11/Gpx4 signaling axis.

PD, a glucoside derivative of resveratrol, exhibits superior antioxidant and anti-inflammatory properties compared to resveratrol (Karami et al., 2022; Zhao et al., 2022). PD’s efficacy has been proven in clinical trials and it can be a good choice for several diseases, such as chronic pelvic pain, irritable bowel syndrome (IBS), liver disease, and drug-related rash. Various new drug delivery systems such as nanoparticles, liposomes, micelles, quantum dots, and polymeric nanocapsules are designed to improve PD’s pharmacodynamics and pharmacokinetics (Karami et al., 2022). As a natural compound with well-studied biochemical characteristics, including drug toxicity, PD’s therapeutic potential for UC may be more readily applicable in clinical settings than other ferroptosis inhibitors.

Presently, scant evidence exists regarding the protective effects of PD on ulcerative colitis UC. The evidence gathered by certain researchers implies that PD can mitigate inflammation in arthritis (Yang et al., 2019; Du et al., 2023). In clinical practice, a study enrolling 54 patients with irritable bowel syndrome (IBS) and 12 healthy controls indicated that dietary supplementation of palmithoylethanolamide/polydatin had a notable impact on abdominal pain in IBS patients (Cremon et al., 2017). Previous investigations have shown that PD treatment can inhibit inflammation and preserve the integrity of the intestinal epithelial barrier both *in vitro* and *in vivo* (Yao et al., 2011; Chen et al., 2021). Similarly, our *in vitro* findings revealed that PD treatment lowered inflammatory factor release while enhancing intestinal epithelial barrier function by maintaining the level of TJ protein ZO-1 and occludin, suggesting its potential to mitigate injury caused by DSS-induced colitis. Distinctively, we, for the first time, explored the protective effect of PD on UC from the perspective of the mechanism of inhibiting ferroptosis.

Since iron metabolism, oxidative-reductive pathways, and lipid metabolism jointly regulate ferroptosis, modifying these three pathways through genetic and pharmacological means has been demonstrated to have an impact on ferroptosis (Sun et al., 2023). Elevated levels of reactive oxygen species (ROS) can lead to DNA damage, protein denaturation, and lipid peroxidation through the actions of cyclooxygenase (COX), lipoxygenase (LOX), and other enzymatic processes in UC. Ferroptosis, resulting from the lethal accumulation of lipid-based ROS, is regarded as a significant pathophysiological mechanism underlying UC. Therefore, we hypothesized that the oxidative-reduction pathways, as a common overlapping pathway for ferroptosis and UC, is likely the mechanism by which PD protects UC. And our bulk RNA-seq and pathways enrichment analysis outcomes provided the possible pathway cues which just match the oxidative-reductive pathways.

Ferroptosis, an iron-dependent and caspase-independent nonapoptotic cell death, is predominantly mediated by iron metabolism and lipid peroxidation signaling. The biological attributes of ferroptosis are characterized by the aggregation of iron and ROS, which impair the function of system Slc7a11 and Gpx4 by decreasing cystine uptake and exhausting glutathione (GSH) levels (Li and Huang, 2022). Recent studies have noted that PD can inhibit ferroptosis to ameliorate brain injury (Huang et al., 2021) and acute kidney injury (Zhou et al., 2022), although the detailed mechanisms were not comprehensively discussed. Our findings demonstrated that treatment with PD significantly inhibited mitochondrial ferrous ion accumulation, as well as MDA and ROS production while enhancing SOD activity, suggesting that PD effectively suppresses ferroptosis and lessens inflammation associated with colitis. In terms of phytochemicals, PD is recognized as a strong anti-inflammatory secondary metabolite derived from plants (Mikulski and Molski, 2010), beneficially promoting miR-200a expression to regulate the Kelch-like ECH-associated protein 1 (Keap1)/Nrf2 antioxidant axis. It appears that PD may regulate the expression of Nrf2, influence pathways related to ferroptosis to exert anti-inflammatory capacity.

Researches have shown that the activation of Nrf2 by PD exerts protective effects in various conditions, including liver inflammation (Zhao et al., 2018), spinal cord ischemia/reperfusion injury (Zhan et al., 2021), stress-induced depression (Wang et al., 2023), diabetic neuropathy (Bheereddy et al., 2021), acute myocardial infarction (Chen et al., 2019), etc. For instance, Chen et al. considered that PD mitigates DSS-induced colitis injury may related to alterations of the NF-κB p65, mitogen-activated protein kinases (MAPKs), and AKT/Nrf2/HO-1/NQO1 expression levels, but further evidence is lacking (Chen et al., 2021). Profound investigation is needed to elucidate the role of PD in DSS-induced colitis comprehensively. Our study posited that the protective effect of PD may be linked to reduced ferroptosis via activation of the upstream Nrf2/Slc7a11/Gpx4 signaling pathway. Notably, RSL-3 and erastin, the first two identified ferroptosis-inducing agents, initiate this process by inhibiting Gpx4 and Slc7a11 respectively; both are downstream targets regulated by Nrf2 (Dolma et al., 2003; Yang and Stockwell, 2008; Dodson et al., 2019). We observed that PD significantly upregulates Nrf2 expression and facilitates its nuclear translocation. Subsequently, we employed ferroptosis inducers and inhibitors to assess how modulation of ferroptosis by PD treatment confers protection. Erastin, an inhibitor of Slc7a11, exacerbated ROS levels and mitochondrial iron accumulation during colitis while negating the protective effects conferred by PD *in vitro*. Conversely, Fer-1, a Slc7a11 inducer, reduced intestinal inflammation levels with effects comparable to those observed with PD in our study. The increase in Gpx4 and Slc7a11 levels induced by PD suggested a mechanism for inhibiting ferroptosis consistent with antioxidant enzyme induction mediated by Nrf2. Typically, Nrf2 is unstable and is easily biodegradable (Torrente and DeNicola, 2022; Lee and Roh, 2023). As a prominent negative regulator of Nrf2, Keap1 forms conjugates with Nrf2 which prompt its ubiquitination followed by proteasomal degradation. However, under oxidative stress conditions, Keap1 becomes inactive leading to dissociation from Nrf2; this allows stable translocation of Nrf2 into the nucleus where it activates transcriptional programs targeting genes involved in regulating the Slc7a11/cysteine/glutathione (GSH) axis critical for inhibiting ferroptosis (Jy et al., 2019). Therefore, our research findings provide a more in-depth explanation of the specific mechanisms by which PD protects DSS-induced UC.

The protective role of PD for DSS-induced UC by inhibiting ferroptosis might provide a new rationale for disease incidence and therapeutic approach. It should be noted that this study has merely focused on animal models and cell lines. More attention should be paid to investigate their practical clinical values. Not with standing its limitation, this study does suggest a new possible target and drug for the treatment of UC. Based on our findings, future studies should investigate the synergistic effects of PD with existing UC treatments such as 5-aminosalicylic acid drugs, thiopurines, and biologics. Although mice with DSS-induced colitis display similar clinical symptoms and are commonly utilized for investigating new drugs, extracts, single compounds, and mechanistic studies related to colitis (Chassaing et al., 2014), UC is, after all, a chronic inflammatory bowel disease, and long-term outcomes *in vivo* ought to be examined as well.

In conclusion, our current study suggested that PD holds promise as an adjuvant therapy for the management of intestinal inflammation in colitis. We observed that PD mitigates intestinal inflammation by inhibiting ferroptosis, a process whose suppression is contingent upon the stability of the Nrf2/Slc7a11/Gpx4 signaling axis. The precise mechanisms underlying this phenomenon warrant further investigation. The findings presented herein indicated that PD may serve as a potential therapeutic agent for patients with UC.

## Data Availability

The original contributions presented in the study are publicly available. This data can be found here: https://www.ncbi.nlm.nih.gov/geo/query/acc.cgi?acc=GSE285956.
