# Supplementary material for: Polydatin protects against DSS-induced ulcerative colitis via Nrf2/Slc7a11/Gpx4-dependent inhibition of ferroptosis signalling activation
Source: Front Pharmacol. 2025 Jan 14;15:1513020. doi: 10.3389/fphar.2024.1513020 (PMC11772288; doi:10.3389/fphar.2024.1513020)
Supplement: Supplementary file 3 [file Table1.docx]

| Table S1. The sequence of primers used for RT-PCR | | |
| --- | --- | --- |
| IL-1 β | 5’-CTCGCCAGTGAAATGATGGCT-3’ | 5’­-GTCGGAGATTCGTAGCTGGAT-3’ |
| TNF-α | 5’-TGGGGTTTGTGAAACTGTGA-3’ | 5’-GTTCCTGCACATTCCCTCTC-3’ |
| IL-6 | 5’-AGGAGACTTGCCTGGTGAAA-3’ | 5’-CAGGGGTGGTTATTGCATCT-3’ |
| H-GAPDH | 5’-CTGTTCGACAGTCAGCCGCATC-3’ | 5’-GCGCCCAATACGACCAAATCCG-3’ |
| Abbreviation: RT-PCR, reverse-transcription polymerase chain reaction. | | |
