# Supplementary material for: Polydatin protects against DSS-induced ulcerative colitis via Nrf2/Slc7a11/Gpx4-dependent inhibition of ferroptosis signalling activation
Source: Front Pharmacol. 2025 Jan 14;15:1513020. doi: 10.3389/fphar.2024.1513020 (PMC11772288; doi:10.3389/fphar.2024.1513020)

Fig1D DSS + Polydatin Occludin

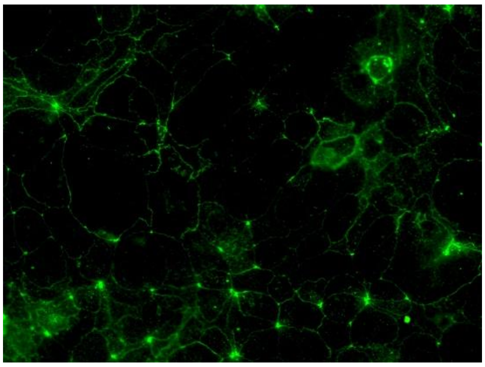

Fig1D DSS + Polydatin Occludin

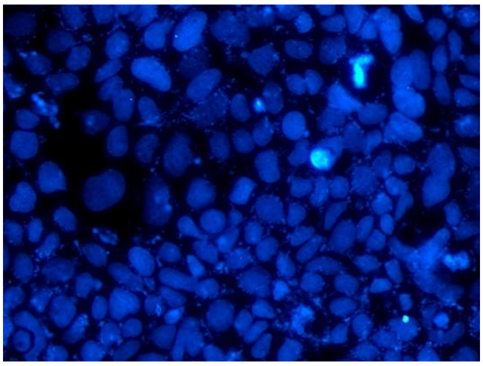

Fig1D DSS + Polydatin ZO1

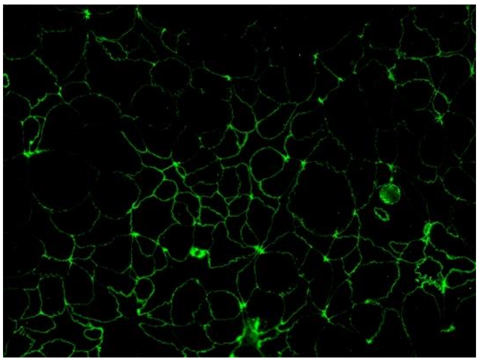

Fig1D DSS + Polydatin ZO1

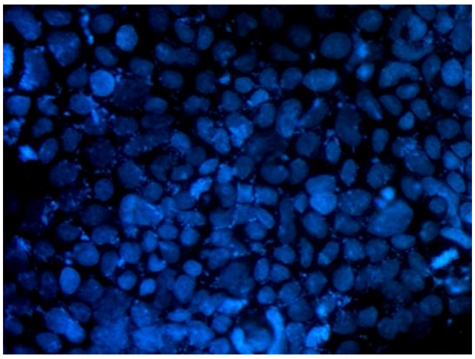

Fig1D DSS Occludin

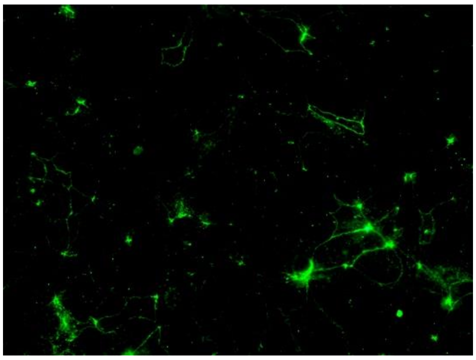

Fig1D DSS Occludin

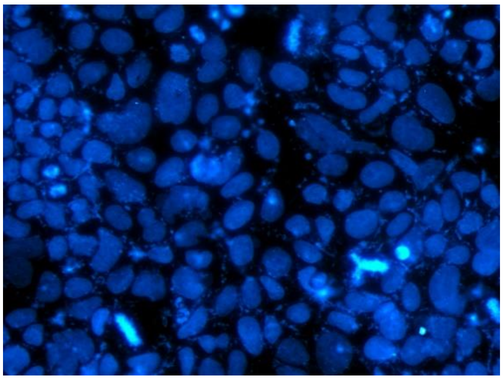

Fig1D DSS ZO1

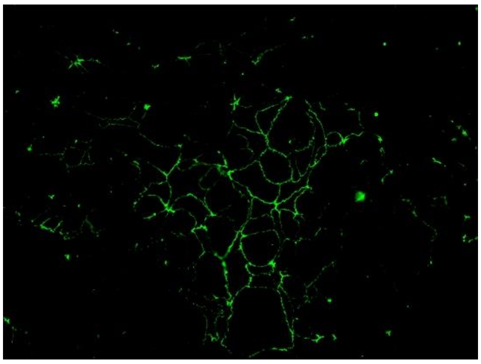

Fig1D DSS ZO1

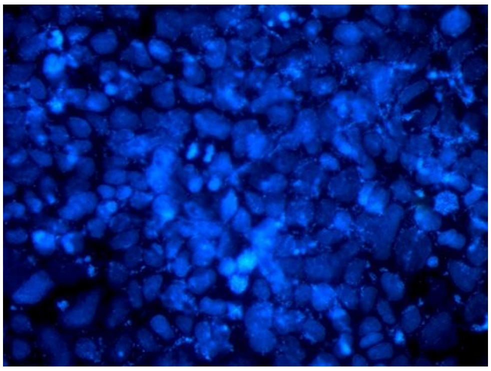

Fig1D NC Occludin

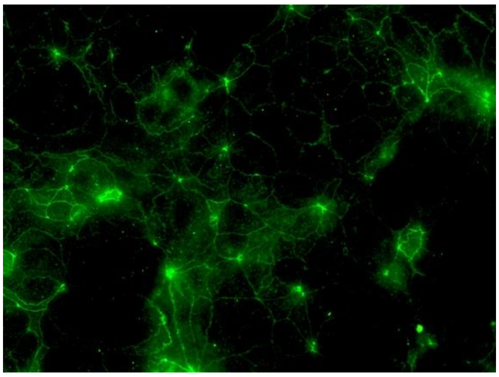

Fig1D NC Occludin

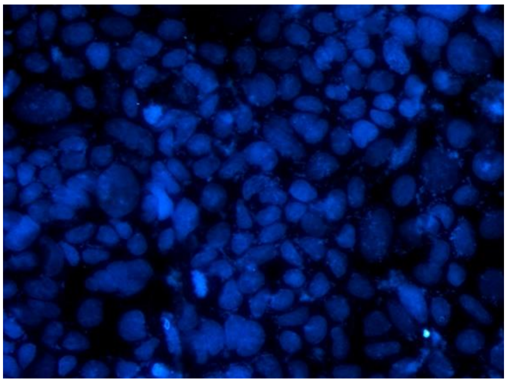

Fig1D NC ZO1

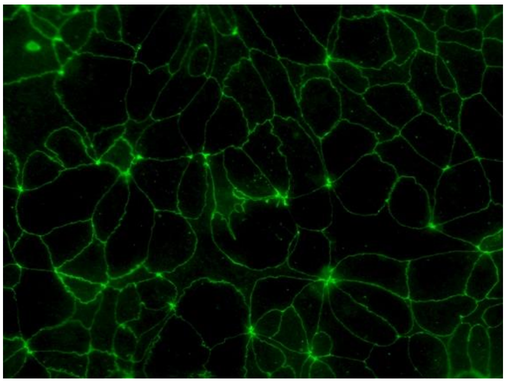

Fig1D NC ZO1

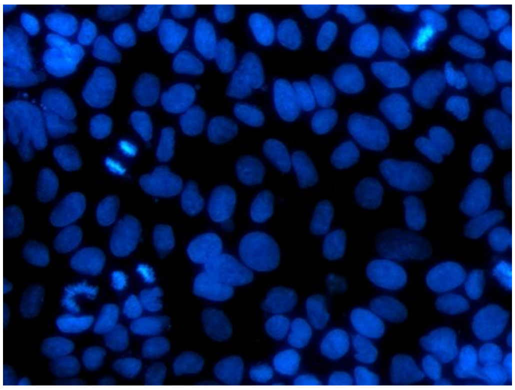

Fig1E WB

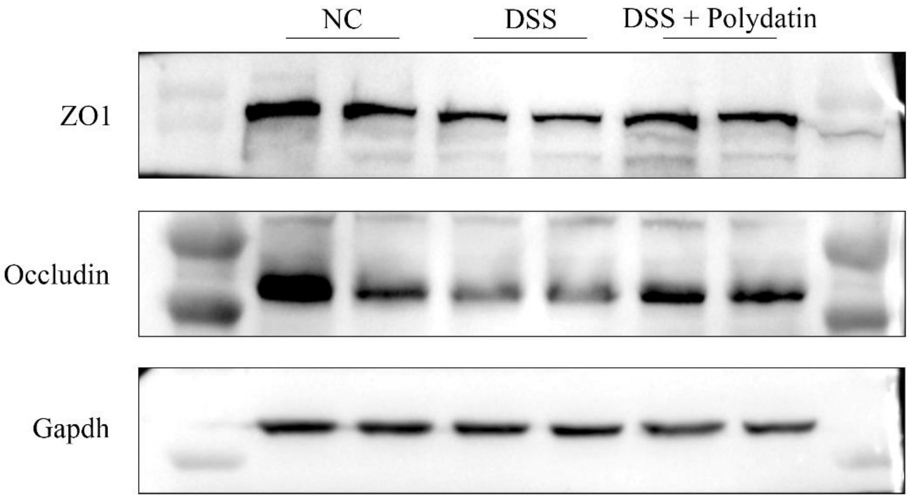

Fig 2G DSS+PD

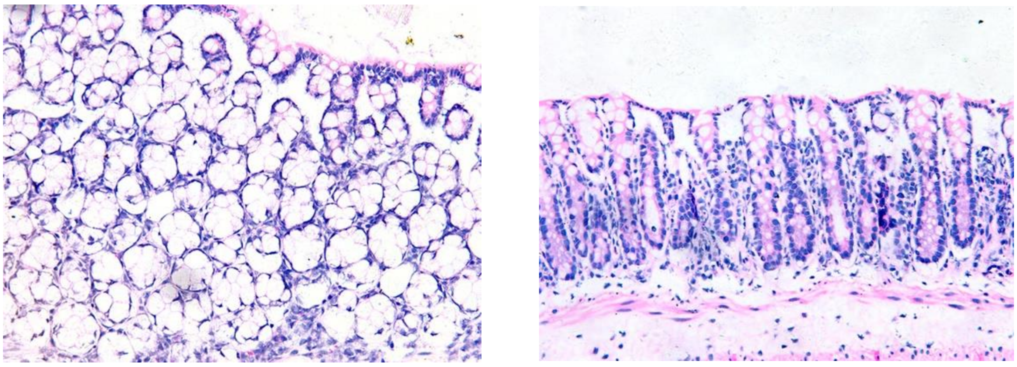

Fig 2G DSS

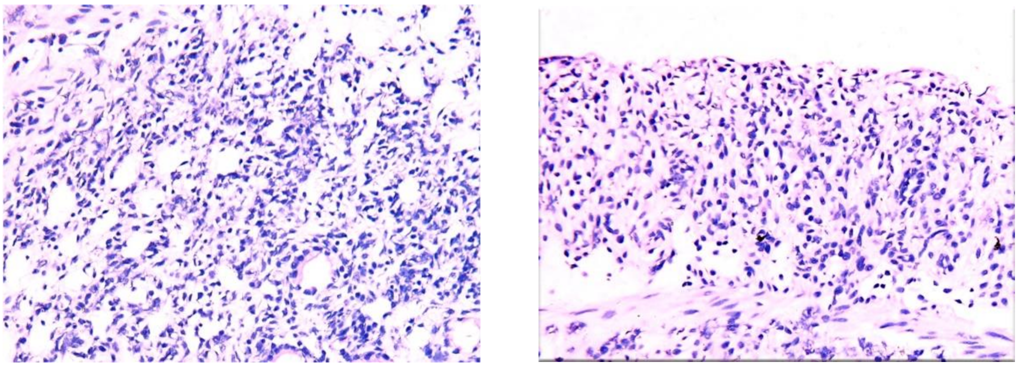

Fig 2G NC

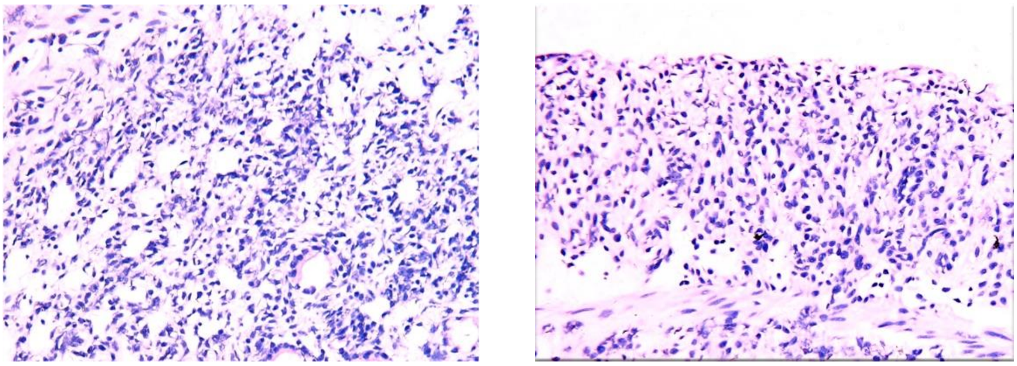

Fig 2I Occludin (DSS+PD)

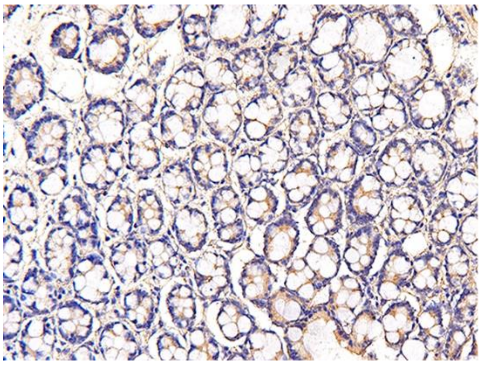

Fig 2I Occludin (DSS)

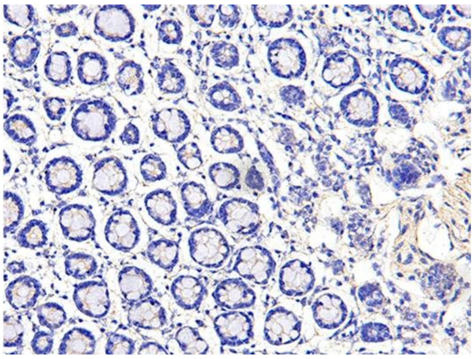

Fig 2I Occludin (NC)

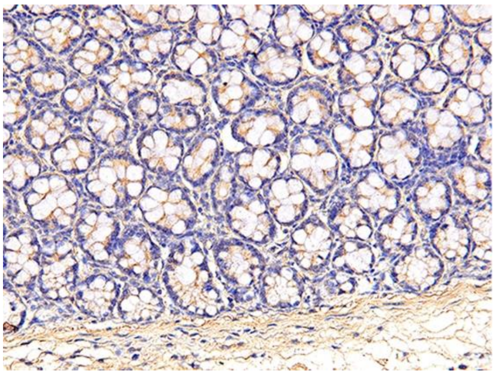

Fig 2I ZO1 (DSS+PD)

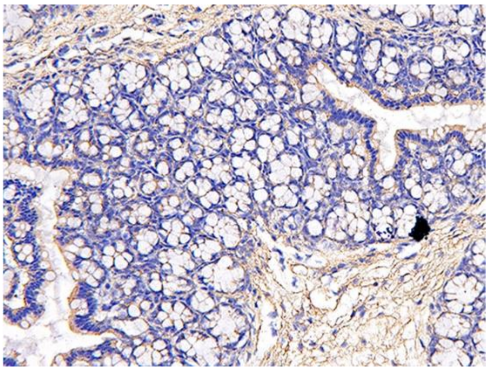

Fig 2I ZO1 (DSS)

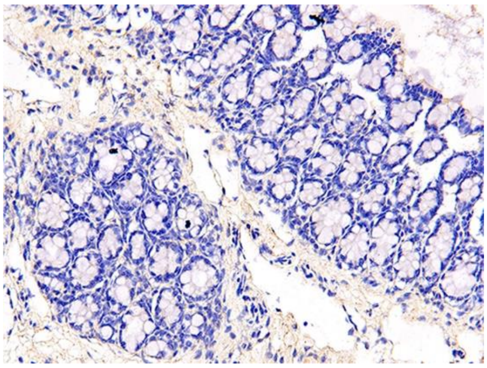

Fig 2I ZO1 (NC)

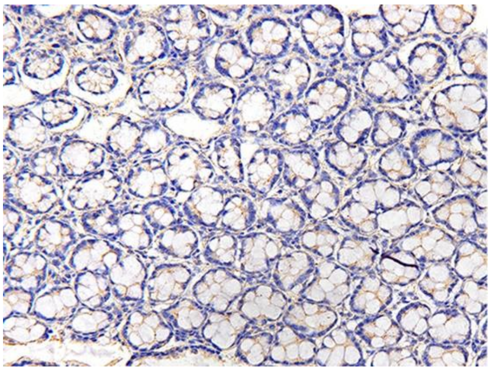

Fig 4A WB

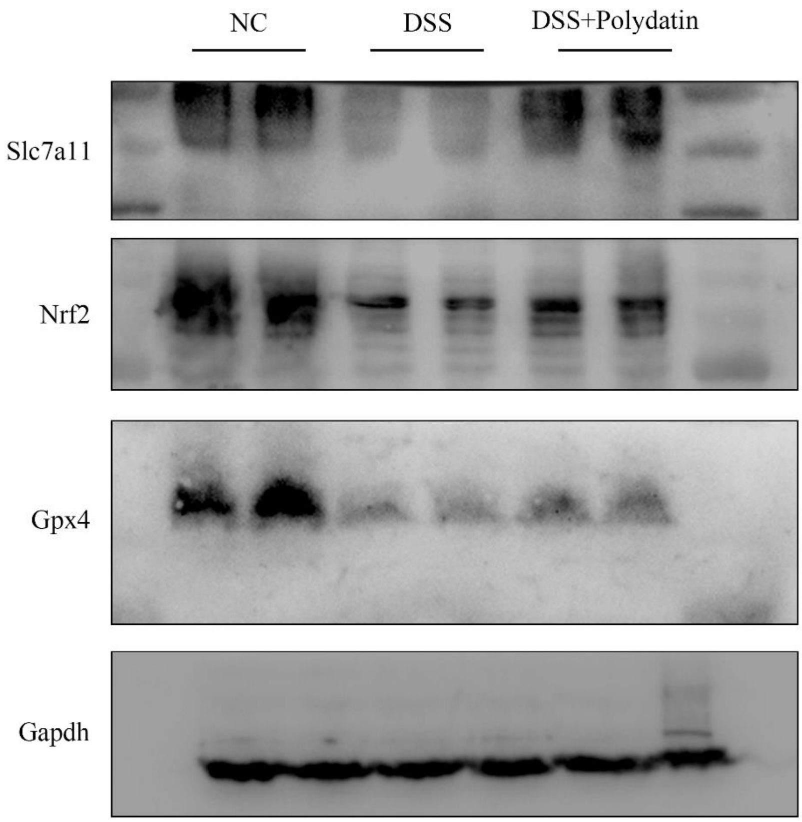

Fig 4C DPAI (DSS+PD)

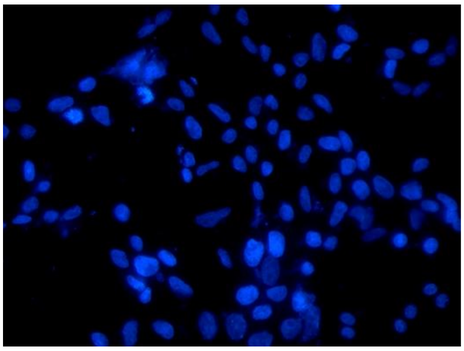

Fig 4C Gpx4 (DSS+PD)

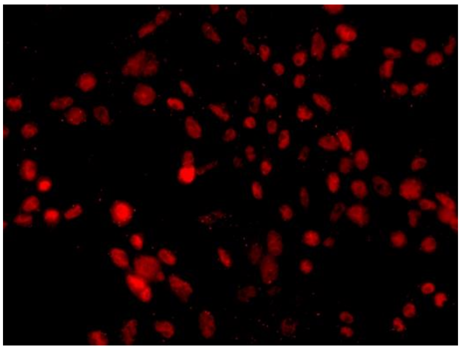

Fig 4C Nrf2 (DSS+PD)

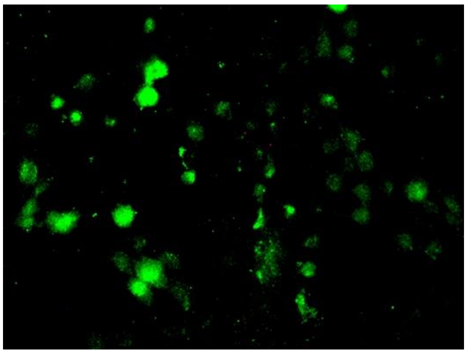

Fig 4C DPAI (DSS)

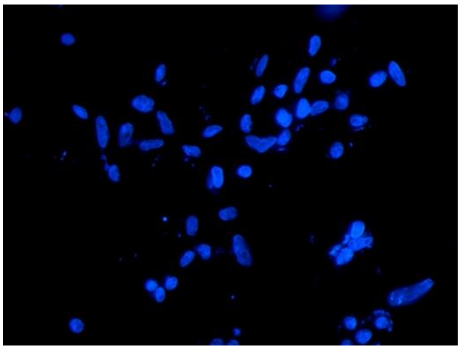

Fig 4C Gpx4 (DSS)

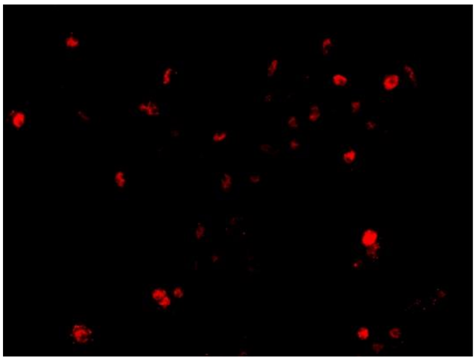

Fig 4C Nrf2 (DSS)

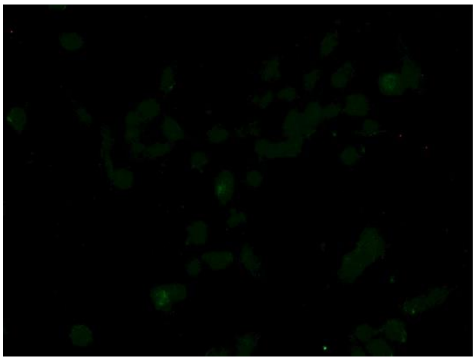

Fig 4C DPAI (NC)

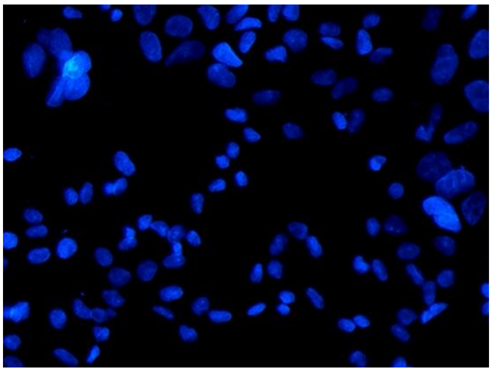

Fig 4C Gpx4 (NC)

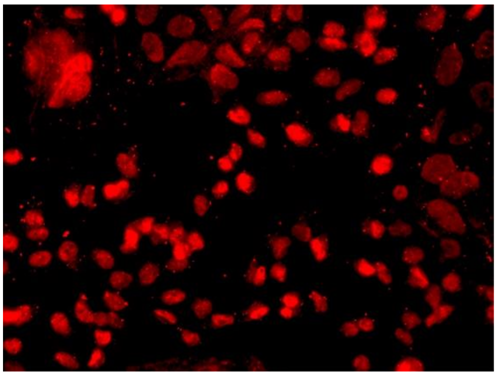

Fig 4C Nrf2 (NC)

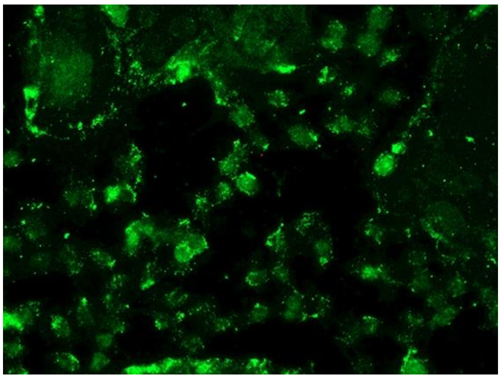

Fig 4F DSS+PD

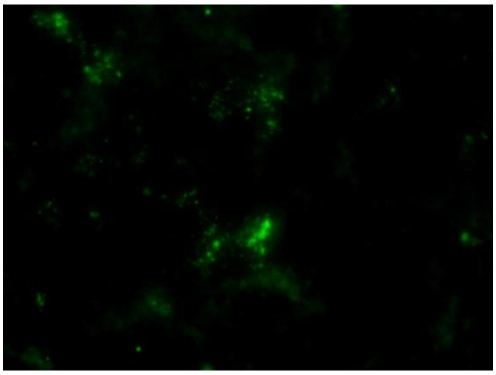

Fig 4F DSS

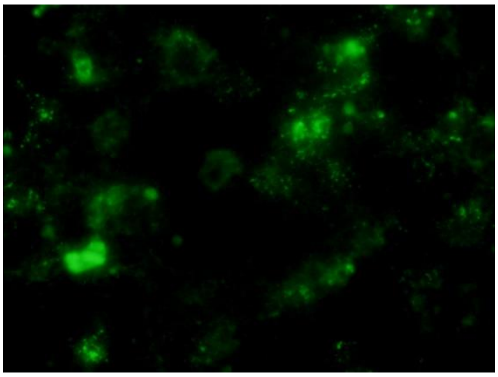

Fig 4F NC

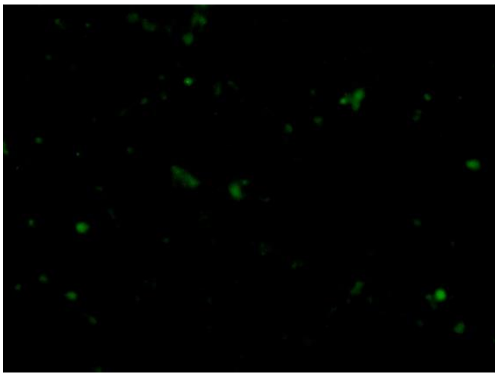

Fig 4G DSS+PD

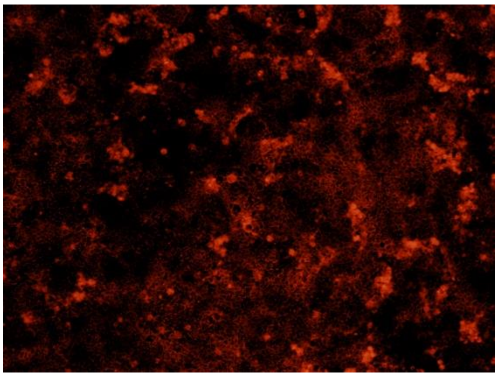

Fig 4G DSS

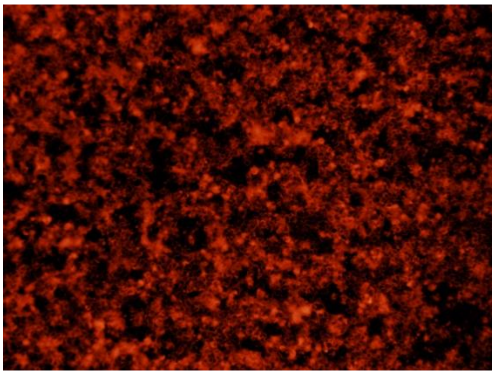

Fig 4G NC

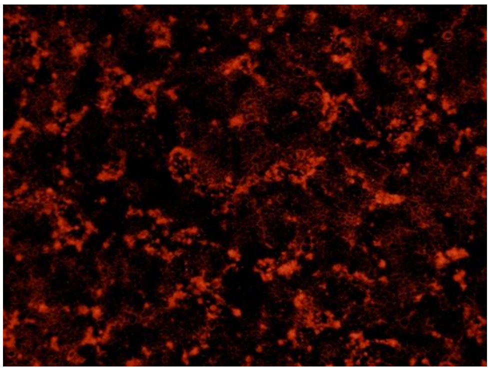

Fig 5A DSS+PD

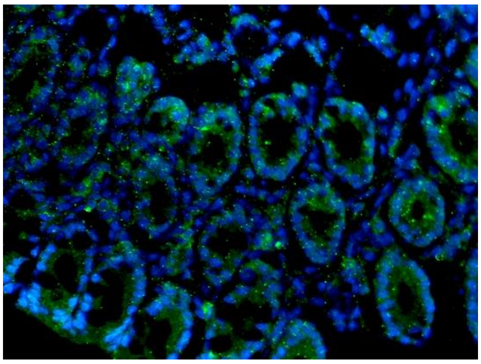

Fig 5A DSS

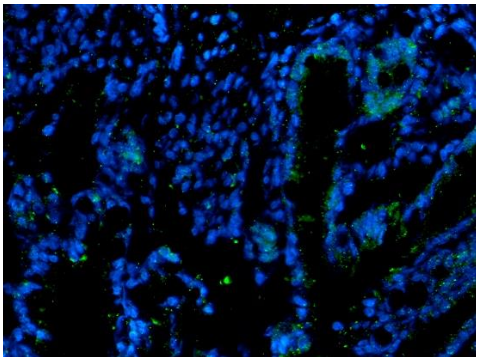

Fig 5A NC

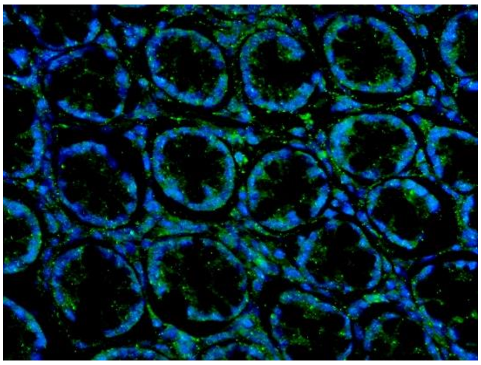

Fig 5B DSS+PD

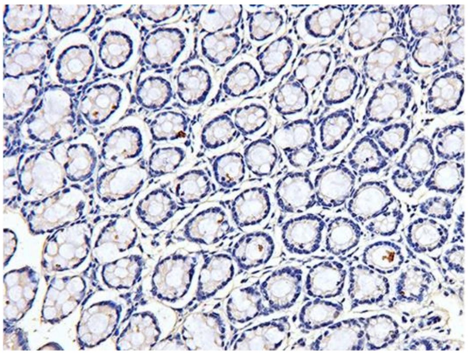

Fig 5B DSS

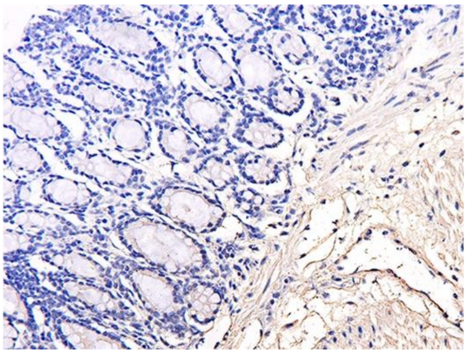

Fig 5B NC

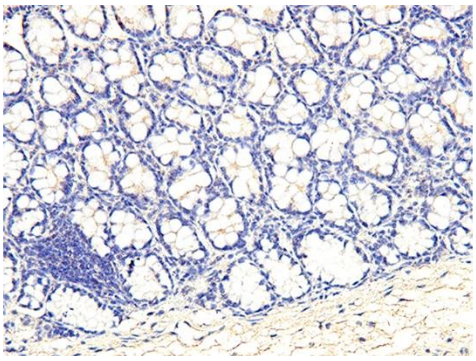

Fig 5C DSS+PD

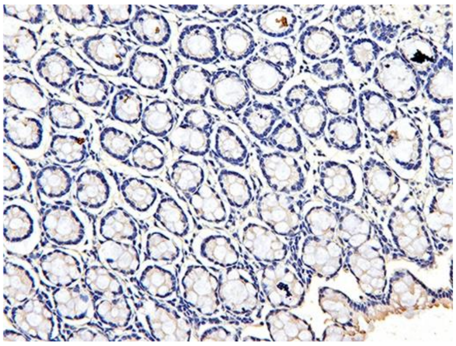

Fig 5C DSS

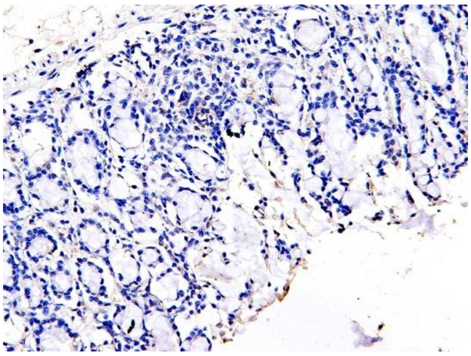

Fig 5C NC

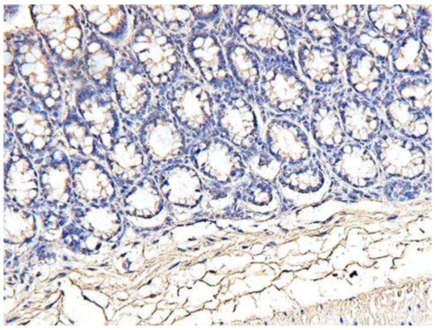

Fig 5D DSS+PD

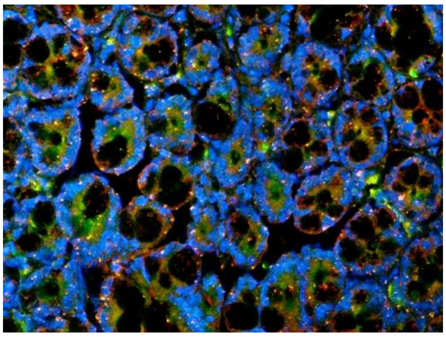

Fig 5D DSS

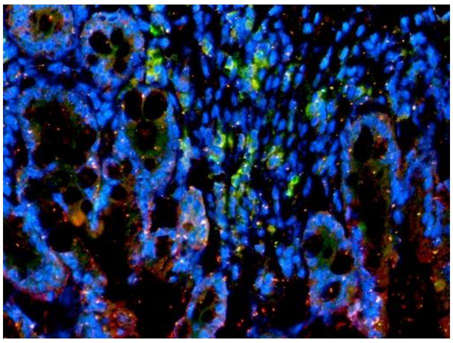

Fig 5D NC

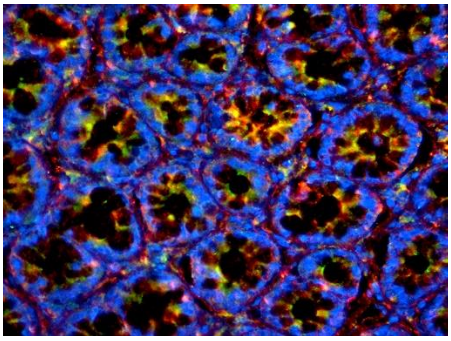

Fig 6A WB

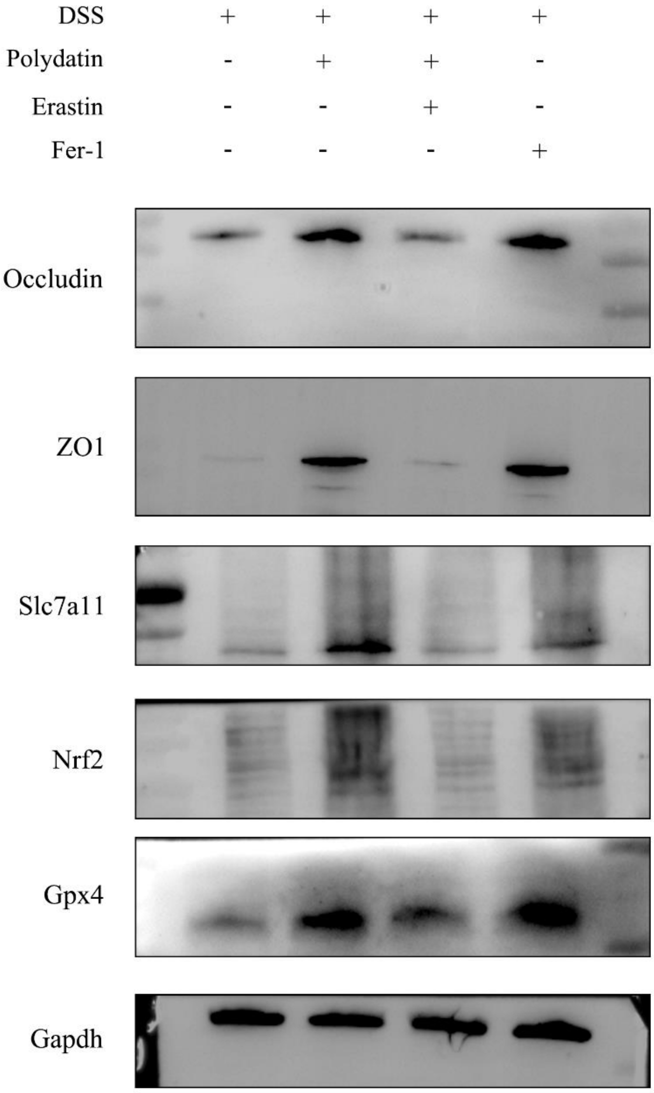

Fig 6C DAPI (DSS)

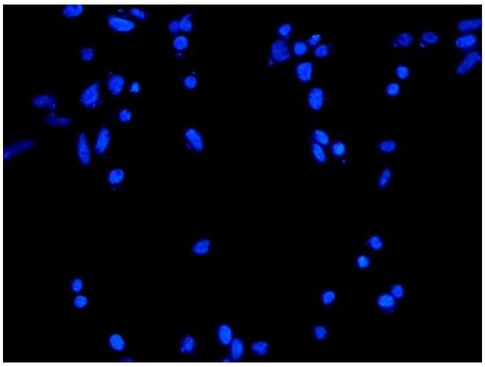

Fig 6C Gpx4 (DSS)

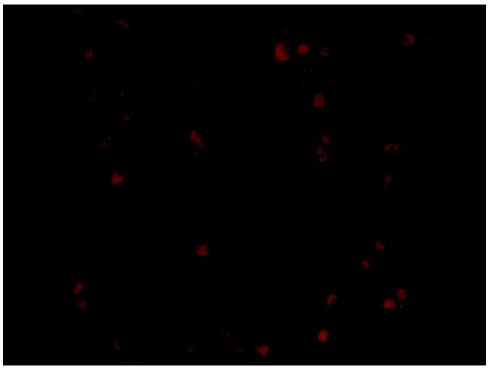

Fig 6C Nrf2 (DSS)

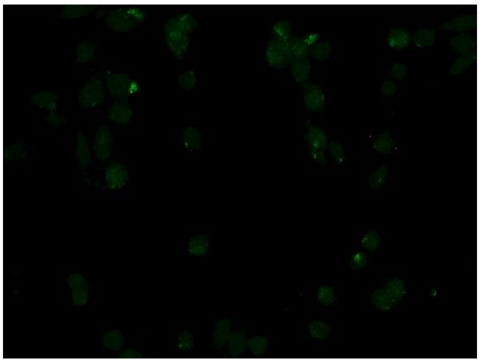

Fig 6C DAPI (DSS+Fer-1)

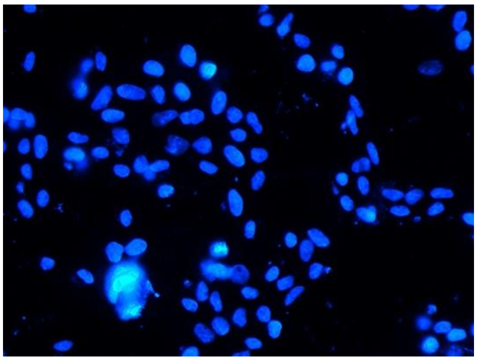

Fig 6C Gpx4 (DSS+Fer-1)

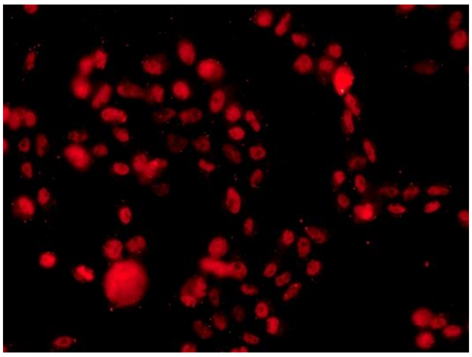

Fig 6C Nrf2 (DSS+Fer-1)

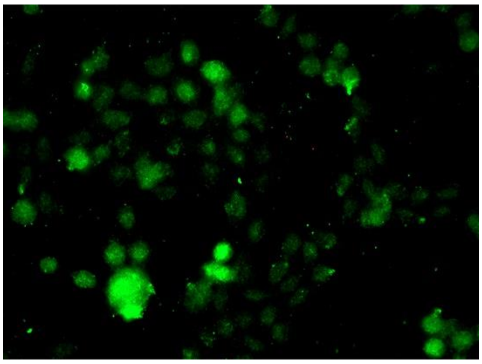

Fig 6C DAPI (DSS+PD)

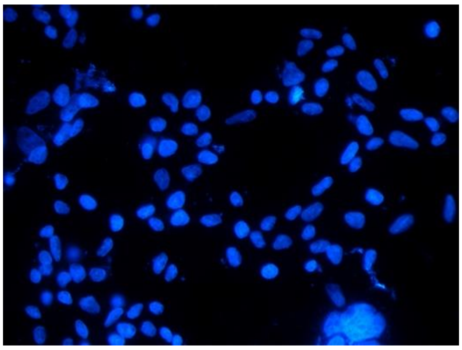

Fig 6C Gpx4 (DSS+PD)

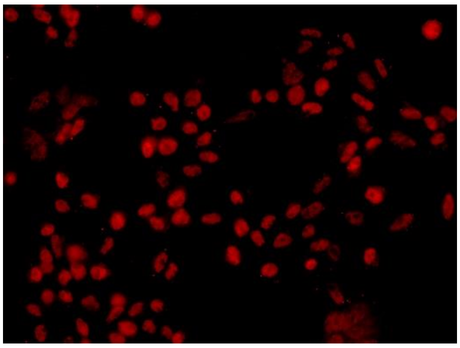

Fig 6C Nrf2 (DSS+PD)

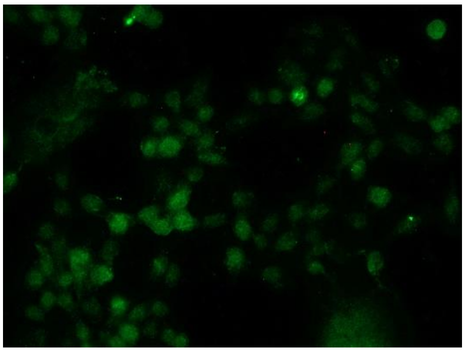

Fig 6C DAPI (DSS+PD+Erastin)

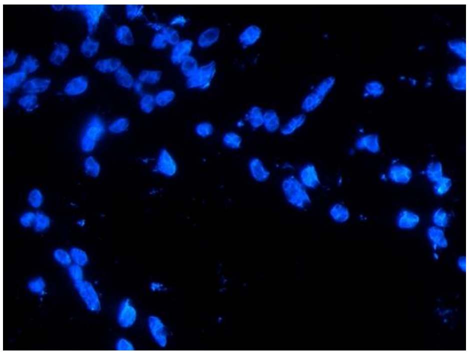

Fig 6C Gpx4 (DSS+PD+Erastin)

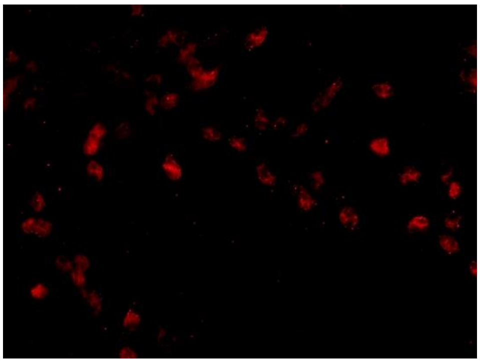

Fig 6C Nrf2 (DSS+PD+Erastin)

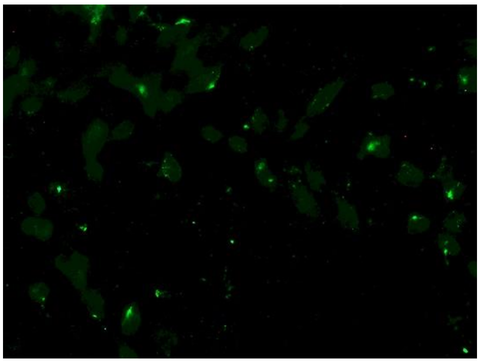

Supplement: Supplementary file 5 [file DataSheet1.pdf]
